# A Gain-of-Function Mutant of IAA7 Inhibits Stem Elongation by Transcriptional Repression of *EXPA5* Genes in *Brassica napus*

**DOI:** 10.3390/ijms22169018

**Published:** 2021-08-21

**Authors:** Tao Wei, Li Zhang, Ruijia Zhu, Xuefei Jiang, Chu Yue, Ying Su, Hongpei Ren, Maolin Wang

**Affiliations:** Key Laboratory of Bio-Resource and Eco-Environment of Ministry of Education, College of Life Sciences, Sichuan University, No. 24 South Section 1, Yihuan Road, Chengdu 610065, China; 2017322040057@stu.scu.edu.cn (T.W.); 2019222040121@stu.scu.edu.cn (L.Z.); zrj0323@stu.scu.edu.cn (R.Z.); 2018322040036@stu.scu.edu.cn (X.J.); 2019222040110@stu.scu.edu.cn (C.Y.); 2020222040084@stu.scu.edu.cn (Y.S.); 2020222040115@stu.scu.edu.cn (H.R.)

**Keywords:** *Brassica napus*, plant height, Aux/IAA, ARF, EXPANSIN

## Abstract

Plant height is one of the most important agronomic traits of rapeseeds. In this study, we characterized a dwarf *Brassica napus* mutant, named *ndf-2*, obtained from fast neutrons and DES mutagenesis. Based on BSA-Seq and genetic properties, we identified causal mutations with a time-saving approach. The *ndf-2* mutation was identified on chromosome A03 and can result in an amino acid substitution in the conserved degron motif (GWPPV to EWPPV) of the Auxin/indole-3-acetic acid protein 7 (BnaA03.IAA7) encoded by the causative gene. Aux/IAA protein is one of the core components of the auxin signaling pathway, which regulates many growth and development processes. However, the molecular mechanism of auxin signal regulating plant height is still not well understood. In the following work, we identified that BnaARF6 and BnaARF8 as interactors of BnaA03.IAA7 and *BnaEXPA5* as a target of BnaARF6 and BnaARF8. The three genes *BnaA03.IAA7*, *BnaARF6/8* and *BnaEXPA5* were highly expressed in stem, suggesting that these genes were involved in stem development. The overexpression of *BnaEXPA5* results in larger rosettes leaves and longer inflorescence stems in *Arabidopsis thaliana*. Our results indicate that BnaA03.IAA7- and BnaARF6/8-dependent auxin signal control stem elongation and plant height by regulating the transcription of *BnaEXPA5* gene, which is one of the targets of this signal.

## 1. Introduction

Plant height is a key agronomic trait closely related to plant architecture, photosynthetic efficiency and productivity [1,2]. The core contents of the “green revolution” are the introduction of semi-dwarf rice (*Oryza sativa*) and dwarf wheat (*Triticum aestivum*) varieties in agricultural cultivation, which increased harvest index at the expense of straw biomass and significantly improved lodging resistance associated with wind and rain in both crops [3]. Plant growth and development is a very precise and complex regulatory process. Plant height was controlled by genetic and environmental factors [4]. For the genetic factor, it mainly involves phytohormone-related pathways, such as gibberellin (GAs) [5,6], brassinosteroid (BRs) [7], strigolactone (SL) [8], and auxin [9,10] biosynthesis and signal transduction pathways. Mutations occurring in these processes often lead to plant phenotypic alternation.

Stem length plays a major role in determining plant height, the developmental transition from the vegetative to the reproductive stage initiates internodes elongation [11]. The internodes elongation of monocotyledonous rice is different from that of dicotyledonous *Arabidopsis thaliana*. The internodes of rice originate from vegetative shoot apical meristem and the uppermost 4 or 5 internodes elongate when the transition begins, while *A. thaliana* internodes are derived from reproductive shoot apical meristem and all internodes can elongate [11,12]. The number and size of internode cells define internode length. The growth and multiplication of plant cells not only requires the strength and rigidity of the primary cell walls to provide structural support, but also the expansibility enabling the cell wall to be loosely selective in order to allow water absorption and anisotropic cell expansion [13].

The main structure components of plant cell walls are well understood, but the molecular mechanisms of these components’ interaction and cross-link forming primary cell walls with structural strength and extensibility are unknown [14]. The plant cell wall is composed of cellulose and non-cellulosic matrix polymers, such as pectin and xyloglucans in most land plants, and a small amount of protein, like wall-loosening proteins EXPANSIN [15]. Cellulose microfibers are directly contacted with one another or glued together by lateral noncovalent adhesion with a monolayer of xyloglucan. EXPANSIN targeting in these contact regions disrupt the noncovalent binding with a nonenzymatic form, and they might make the surface glucans of cellulose more vulnerable to enzymatic attack by cellulase. Several transcription factors controlling *EXPANSIN* gene expression are reported [16,17,18,19].

Cell wall expansion and overall cell growth are regulated by several factors, including phytohormones. As one of the most important phytohormones, auxin is involved in regulating a vast number of growth and development responses throughout the life of plants. In this pathway, auxin participates in the formation of co-receptor complexes as a molecular glue between auxin receptors TIR1/AFB F-box protein and negative regulator Aux/IAA, which results in ubiquitination degradation of Aux/IAA by 26S proteasome. Release of Aux/IAA inhibition allows ARF regulating gene transcription. Different Arabidopsis Aux/IAA mutants, such as *axr2/iaa7*, *axr5/iaa1*, *axr3/iaa17*, and *shy2/iaa3*, all showed cell expansion defects [20,21,22], indicating that auxin induces cell expansion by degrading Aux/IAAs. Auxin can also change the cell wall structure by inducing the changes of cell wall components [23], activating the expression of cell wall related genes [24], and stimulating the synthesis of proton pump to cause apoplast acidification [25].

Auxin plays a vital regulatory role in plant growth and architecture by promoting cell proliferation, expansion, elongation and differentiation. In Arabidopsis thaliana, mutations in the conserved GWPPV motif of Aux/IAA protein domain II result in various growth and development defects, such as inhibited stem elongation, leaf curl, slow root growth, few lateral roots and weakened gravitropism [20,21,22]. Similar mutations in rice [26] and rapeseeds [27,28] lead to semblable phenotypic modulation. The auxin-response factor ARF6 interacts with two other transcription factors, PIF4 and BZR1, to cooperatively regulate common target genes and cell elongation [29]. In *A. thaliana*, *arf6/arf8* double mutants, *arf6* and *arf8* single mutants had twisted leaves and short inflorescence stems [30,31]. It has been reported that *EXPA* gene overexpression induces large plant cells, larger leaves, longer stems [32,33,34], root growth and lateral root formation [35,36], while its knockdown and silence leads to shorter plants [37], reduced lateral root formation [38] and firmer fruits [39]. In *A. thaliana*, *EXP8* gene expression has been shown to be positively regulated by auxin-responsive gene transcription factor ARF7 [40], which plays an important role in cell extensive growth [41]. However, there are few full well-defined signal transduction networks that explain the process from the initial hormonal receptor to the activation of cellular mechanisms that loosen the cell walls and stimulate cell growth and morphogenesis [38].

In the past decades, although great progress has been made in the exploration of dwarfism and plant type breeding of *Brassica napus*, exploring new germplasm resources is still of great significance to cultivate ideotype rapeseeds with higher yields and elucidate the mechanism of plant development. In the present study, we reported a dwarf mutant of *ndf-2* with a plants height of ~87 cm obtained from fast neutron and diethyl sulfate (DES)-mutagenized doubled haploid (DH) line 3529. *ndf-2* is an excellent germplasm resource for semi dwarf breeding. The plant height of F_1_ generation crossed between 3529 and *ndf-2* ranges from 121 to 167 cm, which meets the ideotype standard of *B. napus* [42]. The objectives of this study were to: (1) identify the gene causing dwarf architecture in *ndf-2* based on BSA-seq and the genetic property; (2) develop a simple and effective molecular marker co-segregated to the causative gene that could be used for marker-assisted selection breeding; and (3) explore the dwarfing molecular mechanism caused by the mutation gene in *ndf-2*. Here, we show that *ndf-2* encodes an Aux/IAA7 (BnaA03.IAA7) protein, and a single amino acid substitution in conserved domain II of this protein resulted in changing the GWPPV degron motif to EWPPV. As BnaA03.IAA7 protein is likely to act as a transcriptional repressor in auxin signal transduction, we also examined the involvement of Aux/IAA- and ARF-dependent signaling in stems elongation of rapeseeds. We identified BnaARF6 and BnaARF8 as interactors of BnaA3.IAA7 and the gene *BnaEXPA5* encoding cell wall loosening protein as a common target of BnaARF6 and BnaARF8. In *Arabidopsis thaliana*, ectopic overexpression of *BnaEXPA5* results in longer stems and larger leaves, contrary to the phenotype of the *ndf-2* and the Arabidopsis *axr2/iaa7* mutant. Our results suggest that *ndf-2*, as a repressor of auxin signal, plays an important role in stem elongation of *B. napus*, which may provide an effective foundation for plant type breeding in *B. napus*. Our findings may also provide new insights for a better understanding of the molecular mechanisms underlying cell wall expansion and stem elongation.

## 2. Results

### 2.1. Morphology and Agronomic Traits of ndf-2

**Figure 1:**
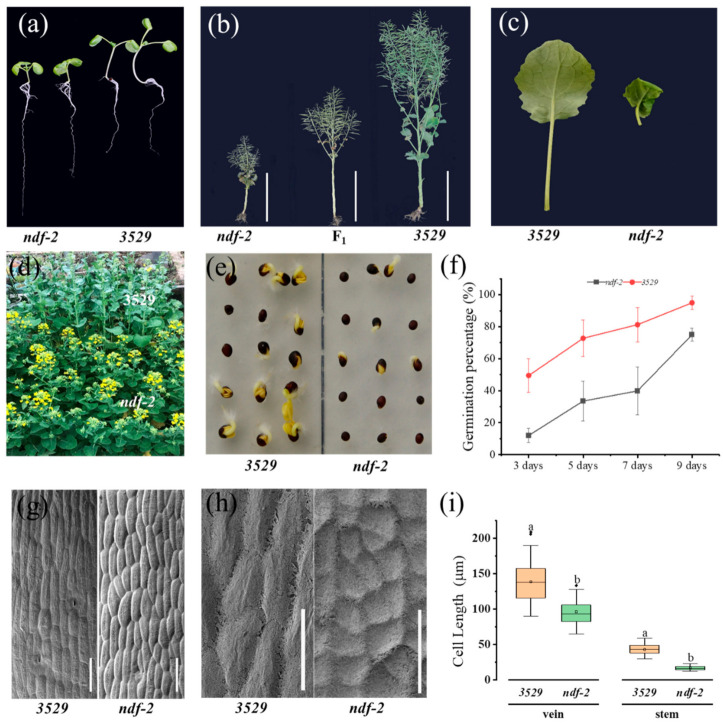
Phenotype of the *ndf-2* mutant of rapeseed. (**a**) Comparison of 1-week-old 3529 (wild-type) and *ndf-2* seedlings. (**b**) Morphology of the 3529 and *ndf-2* at maturation stage. (**c**) Leaf morphology of 5-week-old 3529 and *ndf-2*. (**d**) Difference in flowering time between 3529 and *ndf-2*. (**e**) Seed germination of 3529 and *ndf-2*. (**f**) Comparison of germination rate at 3, 5, 7 and 9 days between 3529 and *ndf-2.* (**g**–**i**) Scanning electron microscopy images of epidermal cells in veins (**g**) and internodes (**h**) of 3529 and *ndf-2*, and quantitative comparison of cell lengths was made in (**i**). Statistically significant differences were revealed using Student’s t-test: *p* < 0.05, different lowercase letters denote significant differences between wild-type and mutant; Bars: (**b**) 50 cm; (**g**) 100 μm; (**h**) 50 μm.

Two dwarf mutants *ndf-1* and *ndf-2* were simultaneously derived from doubled haploid (DH) line 3529 (*Brassica napus*), seeds of which were jointly treated with chemical inducers and bombardment of fast neutron. The principal agronomic characteristics of *ndf-1* and *ndf-2* are presented in Table 1. *ndf-2* showed shorter hypocotyls than 3529 at seedling stage (Figure 1a). At mature stage, the average height of *ndf-2* mutant is 86.8 ± 4.9 cm, which is only 43.7% of its background parent line 3529 (WT) (Table 1, Figure 1b) and slightly higher than the *ndf-1* (Table 1). The dwarfing of *ndf-2* was mainly caused by the shorter internode length. The cells’ length of stem and leaf vein was significantly shorter than that of wild type (Figure 1g–i). It also has dark green, wrinkled, and thickened leaves (Figure 1c). Although *ndf-2* germinated (Figure 1e,f) and grew slowly, its flowering (Figure 1d) and maturation time was about one week earlier than that of wild type 3529. In addition, the 1000 seeds weight of *ndf-2* was higher than that of wild type.

**Figure 2:**
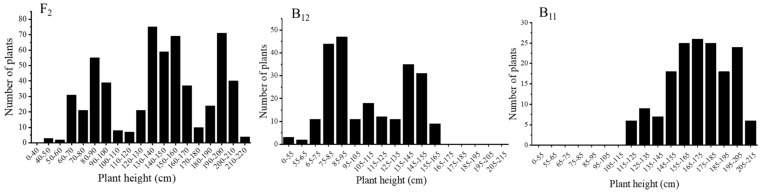
Distribution of plant height in the F_2_ population and backcrosses. F_2_ population (**left**) derived from the cross of *ndf-2* and 3529. B_12_ (**middle**) and B_11_ (**right**) derived from the backcross of F_1_ (*ndf-2* × 3529) with *ndf-2* and 3529, respectively.

The average height of F_1_ plants obtained from the cross of *ndf-2* and 3529 was 152.1 cm, which was close to the intermediate value of *ndf-2* and 3529 plant height. Frequency distributions of the heights of *ndf-2*, 3529, F_2_ and backcrosses are shown in Figure 1. The height in the F_2_ and backcrosses generation presented a continuous and trimodal or bimodal distribution (Figure 2), which corresponded to the phenotypes of two parents and F_1_. According to the results obtained, if defining 3529 genotype as *NDF-2/NDF-2*, dwarf mutant as *ndf-2*/*ndf-2*, then F_1_ generation was *NDF-2/ndf-2*. The separation ratio of F_2_ population was in accordance with 1 (*NDF-2/NDF-2*):2 (*NDF-2/ndf-2*):1 (*ndf-2*/*ndf-2*) (χ^2^ = 3.125, *p* > 0.05), shown in Table 2, and the separation ratios were 1:1:0 for B_11_ and 0:1:1 for B_12_ as determined by Chi-squared tests. These results indicated that the dwarf phenotype of *ndf-2* mutant was controlled by a pair of nuclear gene (*ndf-2*/*ndf-2*).

### 2.2. Aux/IAA-Mediated Auxin Signaling Is Involved in Dwarfism Formation of ndf-2

**Figure 3:**
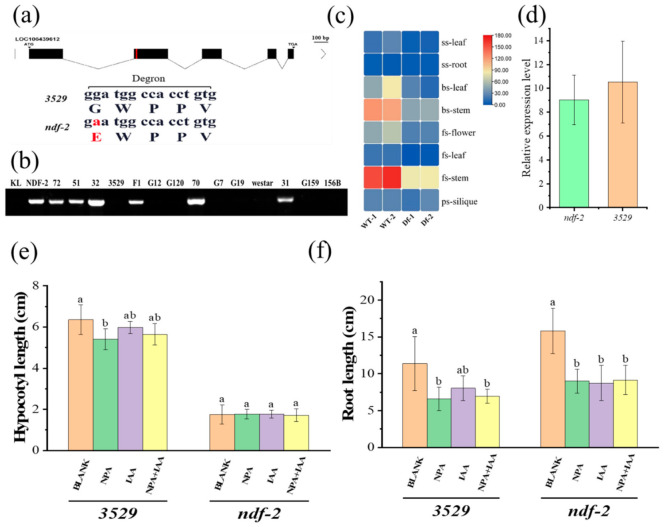
Structure and expression analysis of BnaA03.IAA7 gene and auxin response in 3529 and *ndf-2*. (**a**) Structure of the *NDF-2* gene (*LOC106439612*). A SNP (G–A) in the second exon that converted the conserved Gly to Glu (G84E). Mutated nucleotides and amino acid residues are shown in red. (**b**) Validation of co-segregates between dwarfing phenotype and mutation site. Genotype of *ndf-2*, 3529, F_1_, F_2_ population (‘G + number’ and ‘number’ represent the tall and dwarf plants in the F_2_ population, respectively) and two tall rapeseeds cultivar (westar and KL) were detected using the AS-PCR marker. (**c**,**d**) *NDF-2* gene (*BnaA03.IAA7*) expression levels were compared between *ndf-2* (Df) and 3529 (WT), detected by RNA-seq (**c**) and qPCR (**d**). (**e**,**f**) 1-week-old seedlings of 3529 and *ndf-2* were treated with auxin (IAA; 5 μM), or NPA (0.5 μM) or 5 μM IAA with 0.01 μM NPA for 20 days. Length of hypocotyl (**e**) and root of lateral roots (**f**) are shown, different lowercase letters denote significant differences among the conditions (*p* < 0.05, one-way ANOVA followed by Tukey’s test for multiple comparisons). Values are the means ± SD (*n* = 3).

To identify the gene *ndf-2* that causes plant height reduction and leaf shrinkage mutation phenotype, BSA-seq based on the second-generation sequencing was carried out. Genomic DNA of two F_2_ pools (Df-pool and WT-pool) and two parents pools (*ndf-2*, 3529) was sequenced. SNP was used to calculate the SNP frequency distribution—the SNP index for the Df-pool and WT-pool—and Δ(SNPS-index) was plotted against the genome positions. According to the SNP peak value, the candidate major locus corresponding to dwarfism was identified on A03 chromosome at a 95% confidence interval (Figure S1). By filtering as described in the methods, 83 mutation sites were obtained, and only 6 exon SNPs and InDels were finally identified at the chromosome A03 candidate gene intervals (Table S2). These SNPs and InDels distributed in 6 genes, including an auxin-responsive protein IAA7 encoding gene *LOC106439612 (BnaA03.IAA7)* that was ortholog to *axr2/iaa7* gene in *A. thaliana*. SNP (G251A) on the second exon of *BnaA03.IAA7* results in an amino acid substitution (Gly to Glu) on conserved domain II, which converted the degradation motif from GWPPV to EWPPV (Figure 3a). The other 5 genes (*LOC106438019*, *LOC106386524*, *LOC106389668*, *LOC106376955*, *LOC106442943)* encode uncharacterized protein, glucan endo-1,3-beta-glucosidase A6, glutamine fructical-6-phosphate aminotransferase 2-like, ADP-ribosylation factor GTPase-activating protein (AGD9), and uncharacterized protein, respectively. So far, these five genes have not been reported to be related to plant height.

Genetic transformation confirmed the function of BnaA03.IAA7: one cloned from an EMS mutant [43] which has the same base substitution mutation as *ndf-2*, the other cloned from normal rapeseed performed with site-directed mutagenesis [28]. Both two different amino acid substitutions in the GWPPV motif of BnaA03.IAA7 resulted in short stems and curly leaves. Allele-specific PCR (AS-PCR) molecular markers based on *BnaA03.IAA7* SNP were designed to classify individual types in the F_2_ population. The marker was completely co-segregated with the plant height. All dwarf individuals could amplify out a specific band, but the high F_2_ generation, 3529, 156B and other two high rapeseeds cultivar westar and KL (keleyou1) could not (Figure 3b). This result further proves that *BnaA03.IAA7* is the gene causing dwarf phenotype of *ndf-2*, and the marker can also be used in marker-assisted selection breeding.

To confirm the effect of auxin in the elongation of hypocotyls and roots, 1-week-old 3529 and *ndf-2* seedlings were treated with auxin (IAA; 5 μM) and the polar auxin transport inhibitor N-1-naphthylphthalamic acid (NPA; 0.5 μM) for 20 days. As a result, NPA shortened the hypocotyls and roots of 3529. Moreover, 5 μM IAA also inhibited the hypocotyls and roots elongation of 3529 even in the presence of 0.1 μM NPA (Figure 3e,f). The hypocotyls of *ndf-2* were insensitive to IAA and NPA, but roots elongation was inhibited in the presence of 0.5 μM NPA, 5 μM IAA or both (Figure 3e,f), and more lateral roots were generated (Figure S4).

To further understand the function of BnaA03.IAA7, transcriptomic analyses of *ndf-2* mutant and 3529 wild-type were performed. In seedling stage (ss-root/ss-leaf), booting stage (bs-stem/bs-leaf), flowering stage (fs-stem/fs-leaf/fs-flower) and pods stage (ps-silique), the *BnaA03.IAA7* transcript level of *ndf-2* was not significantly different from 3529, and was mainly expressed in the stem (Figure 3c). Quantitative reverse transcription PCR (qRT-PCR) analyses were consistent with the results in *ndf-2* and 3529 stem (Figure 3d). This suggests that the phenotypic differences between 3529 and *ndf-2* are due to the mutations of *BnaA03.IAA7* rather than transcriptional changes. Subcellular localization experiments showed that the fluorescence of BnaA03.IAA7-GFP fusion protein was present in the nucleus (Figure S2), which was consistent with the function of Aux/IAA protein as a negative regulator of auxin signal pathway.

### 2.3. The Transcription of BnaEXPA5 Is Regulated by Aux/IAA Mediated Signaling Pathway

**Figure 4:**
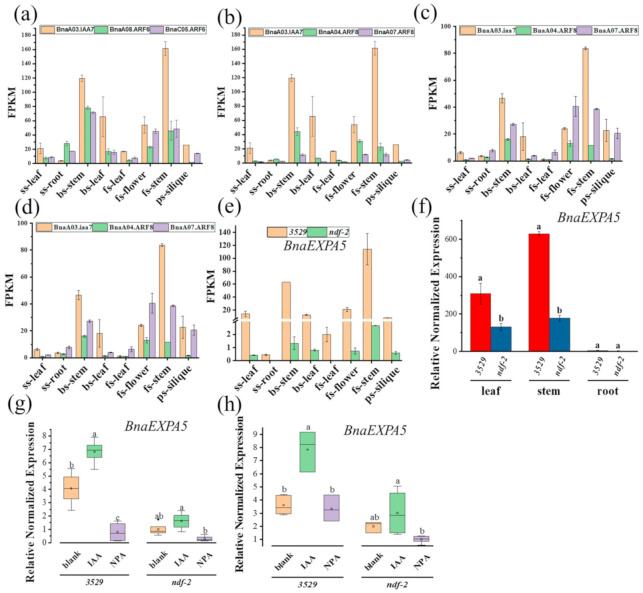
Expression analysis of auxin-related genes in 3529 and *ndf-2* mutants. (**a**–**d**) The expressions of BnaARF6 and BnaARF8 were compared with that of BnaA03.IAA7 in 3529 (**a**,**b**) and *ndf-2* (**c**,**d**). (**e**,**f**) The differential expression of BnaEXPA5 gene between 3529 and *ndf-2* was observed in RNA-seq (**e**) and qPCR (**f**) results. (**g**,**h**) The response of BnaEXPA5 transcription level to IAA or NPA treatment was found in stem (**g**) and leaf (**h**). In (**a**–**e**), the *X*-axis represents the plant tissues of different periods. Values are mean ± SD (*n* = 2 in (**a**–**e**), *n* = 3 in (**f**–**h**). The boxplots show the median (horizontal line), 25% and 75% (down and up edge of the box), minimum and maximum (down and up edge of the whiskers), average (square in the box), and outliers (black spots) (*n* = 4 to 6). Different lowercase letters denote significant differences among the genotypes (**f**) and conditions (**g**,**h**) (*p* < 0.05, one-way ANOVA followed by Tukey’s test for multiple comparisons). Values are the means ± SD (*n* = 3).

In the classical auxin signal transduction network, Aux/IAA proteins interact with ARF proteins to suppress the transcriptional activity of ARF protein. To identify the interactors of BnaA03.IAA7, the transcription level of *ARF* genes in *ndf-2* and 3529 stems was analyzed by transcriptome sequencing. Among all the *ARF* transcripts that has been detected, two *ARF6* genes (*BnaA08g17390D*, *BnaC05g23210D*) and two *ARF8* genes (*BnaA04g07950D*, *BnaA07g25390D*) have similar expression patterns to that of *BnaA03.IAA7* in *ndf-2* mutant and 3529 wild type (Figure 4a–d); they are homologous to the activator *AtARF6* and *AtARF8* genes that have been identified to promote hypocotyl [44] and inflorescence stems elongation [30,31] in *A. thaliana*. In addition, the higher FPKM value (reflecting the relative expression level) of these genes was found in bs-stem and fs-stem (Figure 4a–d).

To identify the genes regulated by Aux/IAA-mediated signaling network, we analyzed the differentially expressed genes (DEGs) in the booting stages stem (bs-stem) and flowering stages stem (fs-stem) between *ndf-2* and 3529 by transcriptome sequencing. A total of 1296 genes had transcription levels at least 8-hold higher (FDR < 0.05) in 3529-bs-stem and 3529-fs-stem than in *ndf-2*-bs-stem and *ndf-2*-fs-stem (Figure S3, Table S3). Among these genes, we focused on the gene *BnaEXPA5 (BnaCnng51340D)*, because some *EXPANSIN* genes were reported to be involved in cell growth and expansion [33] and their expression may be regulated by auxin or ARFs [40]. The transcriptional level of *BnaEXPA5* in 3529 stem was significantly higher than that in *ndf-2*, and similar differences existed in different tissues at other stages (Figure 4e). Interestingly, *BnaEXPA5* also had the highest level of transcript accumulation in the stem, similar to BnaA03.IAA7, *BnaARF6* and *BnaARF8* (Figure 4a–e). qPCR also showed that the expression of *ndf-2* was lower than that of 3529, which was highly expressed in the stem (Figure 4f).

In addition, qPCR results also showed that the transcription level of *BnaEXPA5* gene in the stems and leaves of rapeseed seedlings was increased by auxin treatment, while it was decreased by NPA treatment (Figure 4g,h). As the expression levels in the roots were extremely low, no detectable differences were found. Prediction from TOPCONS software (https://topcons.cbr.su.se/ (accessed on 16 July 2020)) suggested that BnaEXPA5 protein has signal peptides and is localized outside the cell (Figure S6a). The BnaEXPA5-EGFP fusion protein was not observed in the transformed tobacco protoplasts (Figure S6b), but in the leaf epidermal cell outlines of tobacco BnaEXPA5-EGFP protein could be observed (Figure S6c), indicating that BnaEXPA5 is likely to locate on the cell wall. We also investigated whether the expression of *AtEXPA5* gene in *A. thaliana* is regulated by Aux/IAA-mediated signaling. The results showed that compared to the wild-type, the expression of *AtEXPA5* was decreased in *axr2/iaa7* mutant which has an extreme dwarf phenotype (Figure S5a,b), and the expressions of other *AtEXPA* genes including At*EXPA2*, At*EXPA3*, and At*EXPA6*, were also found to be decreased with varying degrees (Figure S5b). Taken together, these results indicate that auxin signaling mediated by BnaA03.IAA7 and BnaARF6/BnaARF8 may regulate the expression of *BnaEXPA5* gene during stem elongation of plant.

### 2.4. BnaARF6/BnaARF8 Protein Interacts with BnaA03.IAA7 Protein and Binds to AuxRE on the Promoter of BnaEXPA5 Gene

**Figure 5:**
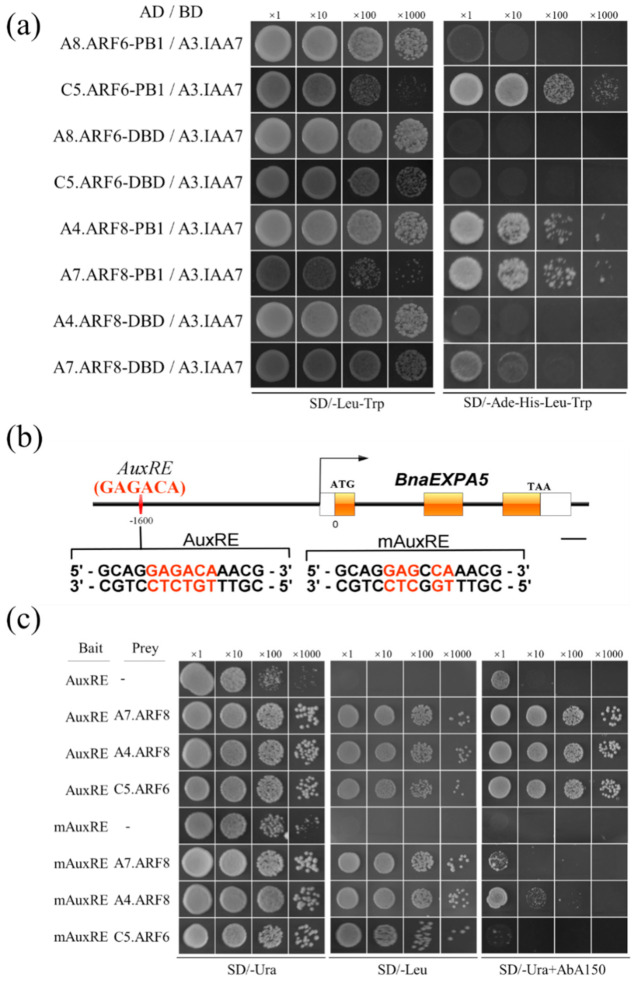
Analysis of BnaARF6/BnaARF8- and BnaA03.IAA7-mediated transcriptional regulation of *BnaEXPA5*. (**a**) Y2H assays to analyze the interaction between BnaARF6/BnaARF8 and BnaA03.IAA7. (**b**) *BnaEXPA5* gene structure and AuxRE cis-element on promoter. The white and orange boxes represent the UTR and exon of the gene, respectively, and the upstream sequence and intron are represented by solid black lines. Sequences of AuxRE and mutant mAuxRE used to construct bait vectors for Y1H are shown, (bar = 200 bp). (**c**) The binding activity of DBD domain of ARF6/ARF8 to AuxRE or mAuxRE was analyzed by Y1H assays. The serially diluted yeast cultures were spotted onto plates SD/−Ura (**left**), SD/−Leu (**middle**) and SD/−Ura + 150 ng/mL AbA (**right**), and growth of the colonies was photographed at 3 days after the spotting. The abbreviations PB1, DBD, A3.IAA7, A8.ARF6, C5.ARF6, A4.ARF8, A7.ARF8 indicate Phox and Bem1 Domain, DNA Binding Domain, BnaA03.IAA7 BnaA08.ARF6, BnaC05.ARF6, BnaA04.ARF8, and BnaA07.ARF8, respectively.

The transcription patterns of *BnaARF6/BnaARF8*, *BnaA03.IAA7* and *BnaEXPA5* genes were well associated with each other, suggesting that BnaA03.IAA7, as a negative regulator, represses auxin signaling by dimerization with the transcriptional activator BnaARF6/BnaARF8, then *BnaEXPA5* would be the target gene of BnaARF6/BnaARF8. This network conforms to the classical auxin signal transduction pathway. To test this hypothesis, the yeast two-hybrid (Y2H) experiments were used to identify the interaction between BnaARF6/BnaARF8 and BnaA03.IAA7. In the Y2H system, BnaA03.IAA7 fuses with the DNA binding domain (BD) of GAL4; DNA-binding domain (ARF-DBD) and Aux/IAA homology domain (ARF-PB1) of *BnaARF6/BnaARF8* fuse with GAL4 activation domain (AD), respectively. Yeast strains (AH109) containing the BD-IAA7 and the AD-ARF-PB1 or the AD-ARF-DBD cassette were generated. The interactions between BnaA03.IAA7 and ARF-DBD or between BnaA03.IAA7 and ARF-PB1 were detected by the growth of yeast on the synthetic dropout minimal base (SD/-His-Leu-Trp-Ade). The results showed that except strains containing BD-IAA7/AD-A8ARF6-PB1, the other four yeast strains containing BD-IAA7/AD-ARF-DBD could grow normally, while all yeast strains containing BD-IAA7/AD-ARF-DBD could not (Figure 5a). These results indicate that BnaA03.IAA7 protein can dimerize with BnaC05.ARF6, BnaA04.ARF8 and BnaA07.ARF8 through the homologous domain PB1, but interacts weakly or not at all with BnaA08.ARF6.

Examination of the *BnaEXPA5* gene promoter region revealed the presence of a single auxin response element AuxRE with the reverse orientation (GAGACA) (Figure 5b), known to bind ARF transcription factors. Furthermore, we performed yeast one-hybrid assays (Y1H) to determine whether the BnaC05.ARF6, BnaA04.ARF8 and BnaA07.ARF8 proteins bind to the element and activate downstream gene expression. The transformed yeast was diluted to the same concentration and grown on a SD/-Ura dropout supplement with the Aureobasidin A (AbA) inhibitory concentration (150 ng/mL). Subsequently, the binding of ARF-DBD to AuxRE or mAuxRE was tested by determining whether yeast can grow normally by activating AbA resistance. The results showed that in the presence of AbA, the yeast strains containing AuxRE and one of three ARF-DBD could grow normally but could not in the strains with mAuxRE. Control strains without prey vector could not grow on both SD/-Leu and SD/-Ura + AbA150 (Figure 5b). All of these results indicate that *BnaEXPA5* gene may be involved in auxin signaling pathway; it was possibly activated by BnaC5.ARF6, BnaA4.ARF8, and BnaA7.ARF8.

### 2.5. Overexpression of BnaEXPA5 Resulted in Longer Stems and Larger Leaves in the Transformed Plants

**Figure 6:**
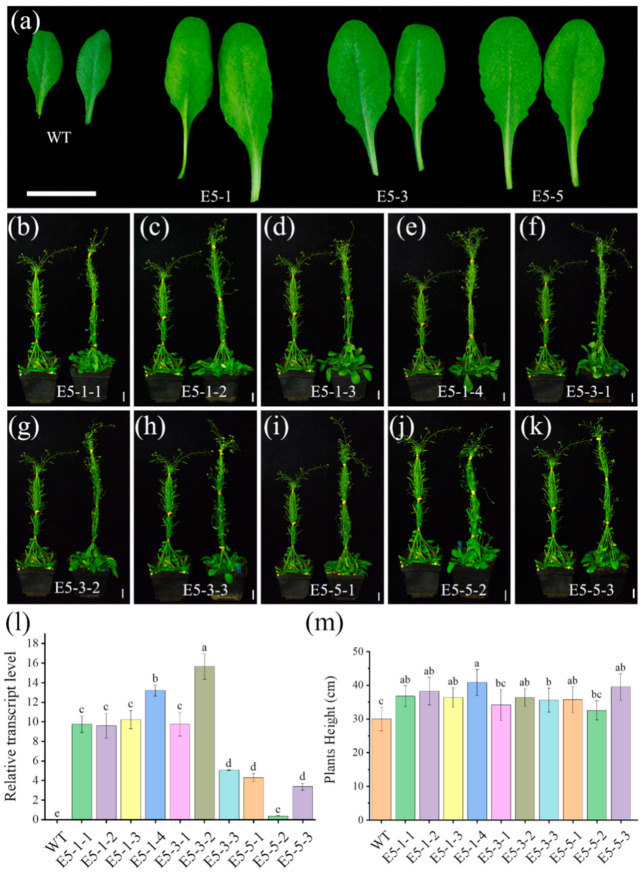
Phenotypic analysis of *BnaEXPA5* transgenic *Arabidopsis thaliana* plants. (**a**) Morphology of the two largest leaves from transgenic lines and WT (wild type), Bar = 2 cm. (**b**–**k**) Comparison of plant height between WT (left in each image) and transgenic plants (right in each image), Bar = 1.5 cm. (**l**) Relative transcript levels of the *BnaEXPA5* from the stems of transgenic plants. The gene *AtUBQ5* served as a reference. Values are mean ± SD (*n* = 3). (**m**) Comparison of the plant heights of the WT and *BnaEXPA5*-transgenic plants. Values are indicated as mean ± SD (*n* > 4). Different lowercase letters denote significant differences among the plant lines (**l**,**m**) (*p* < 0.05, one-way ANOVA followed by Tukey’s test for multiple comparisons).

To evaluate the role of BnaEXPA5 in cell expansion and stem elongation, *BnaEXPA5* gene was overexpressed in WT *Arabidopsis thaliana* (Columbia ecotype). We used the *CaMV 35S* promoter by which *BnaEXPA5* gene can be continuously and stably expressed without regulation by auxin signaling or other factors. The transgenic T1 progenies obtained from Agrobacterium-mediated inflorescence produced T2 plants. A total of three positive lines in the T2 generation (E5-1, E5-3 and E5-5) were obtained. Homozygous transgenic plants (T3) with basta resistance no longer separated were screened for subsequent studies. To evaluate the levels of *BnaEXPA5* expression in the transgenic lines, qRT-PCR primers were designed to amplify the ORF reflecting the transcriptions of the *BnaEXPA5* transgene. The *BnaEXPA5* transcripts in stems and leaves of E5-1, E5-3 and E5-5 were detected. However, there were no detectable *BnaEXPA5* transcripts in WT. There were high and neat transcription levels in the four E5-1 T3 families (E5-1-1, E5-1-2, E5-1-3, E5-1-4), while E5-2-2 families have a relatively low expression level in E5-2 (Figure 6l). These results indicate that *BnaEXPA5* was successfully expressed in *A. thaliana*.

As for the phenotype of transgenic plants, there was no significant difference in hypocotyls, cotyledons and roots of 5-day-old transgenic plants seedlings compared with WT under normal light or dark conditions (Figure S7). However, at the booting stage, in three transgenic lines E5-1, E5-3 and E5-5, rosette leaves with larger area and longer petioles were observed and had stronger growth potential compared to the WT (Figure 6a). At the end of flowering, all transgenic lines except E5-5-2 had higher plant height than WT (Figure 6b–k). The results showed that the mean plant height of all the measured transgenic lines was significantly higher than that of the WT, except E5-5-2 and E5-3-1, which were slightly higher than that of the WT. Although E5-3-2 has the highest expression level of *BnaEXPA5*, the average plant height of this family is not the highest. Meanwhile, although the expression levels of E5-3-3, 5E-5-1, and E5-5-3 were relatively low, their average plant heights are comparable with other transgenic lines. Only E5-5-2 has the lowest expression level and the lowest average plant height. Therefore, we cannot summarize the relationship between the expression level and plant height. Indeed, we proved that *BnaEXPA5* can promote the stem elongation and leaf expansion of *A. thaliana* inflorescence. In addition, no significant effects were observed on other organs, such as flowers and roots (data not shown).

## 3. Discussion

A *Brassica napus* mutant, *ndf-2*, was derived from a high doubled haploid (DH) line 3529, seeds of which were jointly treated with diethyl sulfate and bombardment of fast neutron. Compared with its background parent 3529, the *ndf-2* mutant had decreased plant height, wrinkled leaves (Table 1, Figure 1) and enhanced lodging resistance. The microstructure showed that the cell length of stem and vein of *ndf-2* became shorter than 3529 (Figure 1g–i). Inheritance analysis suggests that the dwarf trait of *ndf-2* was controlled by a single semi-dominant gene *ndf-2* (Table 2, Figure 2). Heterozygote *NDF-2/ndf-2* has semi-dwarf plant height, which is an ideal plant height of rape and suitable for breeding high-quality hybrid rapeseeds varieties. BSA-seq, the combination of bulked segregant analysis (BSA) with whole-genome resequencing, accelerated the identification of QTL in crops, but the result is often a genomic region containing dozens or more genes. Identifying causative gene still requires discovery of more DNA markers in this region and conducting linkage analysis in a large population, which is a time-consuming, expensive and tiring process. Instead of classical marker linkage analysis following primary mapping of BSA-Seq, we proposed a novel strategy for the isolation and identification of causal mutations and candidate genes based on genetic property, phenotyping and SNP genotyping (as described in the Section 4). Using this strategy, a large number of causally impossible SNPs and InDels in the candidate interval (peak region) were filtered out, and then candidate genes were quickly identified without the time-consuming fine-mapping process.

Of the 83 remaining mutation sites, six SNPs and InDels were located on exons and caused changes in the amino acid sequence. We finally identified the causative gene encoding Aux/IAA protein (BnaA03.IAA7) located on chromosome A03. There is a single amino acid substitution in the degron motif GWPPV of domain II (Figure 3a), which is absolutely conserved in plants and plays an important role in the degradation of Aux/IAA protein required for auxin signal transduction. Mutations in this motif have been found to cause plant height defects, including short hypocotyls and dwarfing, as well as curly leaves [20,21,22,26]. The interaction between Aux/IAA inhibitor protein domain II and auxin receptor TIR1/AFB protein induces the degradation of Aux/IAA inhibitor proteins [45]. Mutations in this domain often affect the stability of these proteins and reduce the response to auxin [46,47]. Sequence alignment and truncation experiments showed that there was a 13 amino acid degradation motif in this domain, which was necessary for auxin mediated degradation [46,48,49,50]. The degradation rate of Aux/IAA proteins is not consistent and depends on the degree of matching with the degrader. The higher the matching, the faster the degradation [51,52,53]. Aux/IAA protein with differential degron or complete lack of domain II, had little or no auxin-induced degradation, which confirmed the role of Aux/IAA degradation rate in regulating auxin response [51,52]. This implied that mutation in BnaA03.IAA7 increased the stability of Aux/IAA protein and higher levels of auxin are required to induce degradation, leading to shorter internodes of *ndf-2*, although *BnaA03.IAA7* transcripts levels were similar to 3529 (Figure 3c,d). We also found that auxin transport inhibitor NPA could shorten the hypocotyls and roots of rape seedlings (Figure 3e,f and Figure S4), while *ndf-2* hypocotyls were less sensitive to NPA. In addition, 5 μM IAA inhibited hypocotyl and root elongation even in the presence of 0.1 μM NPA (Figure 3e,f), indicating that there was an optimal auxin level to initiate hypocotyl elongation and cell expansion. The results showed that auxin was a key regulator of growth and plant height formation in *Brassica napus*.

In *B. napus*, we confirmed that one BnaARF6 and two BnaARF8 could interact with BnaA03.IAA7, and gene *BnaEXPA5* encoding cell wall-loosening protein was identified as the common target of BnaARF6 and BnaARF8. It has been reported that ARF6 and ARF8, as well as ARF5, ARF7 and ARF19 are activators of transcription in auxin signaling pathway. Here we demonstrated that BnaA03.IAA7 interacts with BnaC05.ARF6, BnaA04.ARF8 and BnaA07.ARF8 through Aux/IAA domains, but BnaA08.ARF6 does not (Figure 5a). We also found that the DNA binding domain (DBD) of BnaARF6 and BnaARF8 binds to AuxRE of *BnaEXPA5* promoter (Figure 5b,c). This is consistent with the discovery that *ARF7* binds to AuxRE in *EXPA8* promoter in *A. thaliana* [40]. Our results suggest that BnaA03.IAA7 and BnaARF6/BnaARF8 mediated transcriptional regulation of *BnaEXPA5* is involved in stem elongation. These include: (I) the highest accumulation of transcripts of *BnaA03.IAA7*, *BnaARF6*, *BnaARF8* and *BnaEXPA5* in stems and similar patterns of expression (Figure 3c,d and Figure 4a–f); (II) lower expression of *BnaEXPA5* in *ndf-2* and NPA treated WT stems (Figure 4e–h); (III) heterologous expression of *BnaEXPA5* in *Arabidopsis*, stimulating leaf extension and stem elongation (Figure 6).

*BnaA03.IAA7* is a homologous gene of *AXR2/IAA7* of *Arabidopsis thaliana*. *axr2/iaa7* mutant also mutates in the GWPPV motif but has a more severe and extreme dwarfing phenotype than *ndf-2* (Figure S5a), which may be related to the functional differentiation of four BnaIAA7s in *B. napus* and only one IAA7 in *A. thaliana*. Like *BnaA03.IAA7* dwarfing phenotypes, similar *aux/iaa* mutants in *Arabidopsis* and rice also showed inhibition of stem elongation, leaf curling, slow root growth, less lateral roots and weaker geotropism [54,55]. It was found that Aux/IAA polymerization is related to its biological function. The inhibition activity of non-oligomerized Aux/IAA was inhibited [56,57]. The ability of polymerization adds another layer of potential complexity for Aux/IAA to inhibit ARF transcription factors [58]. Yeast two-hybrid experiments showed that BnaA03.IAA7 could polymerize with itself or the other three BnaIAA7s (Figure S7b). BnaARF6 and BnaARF8 can also interact with most of these BnaIAA7s, but the affinity was different (Figure S7a). This suggested that BnaA03.IAA7 and BnaARF6/BnaARF8 interact like a complex network rather than a single pathway, and therefore the regulation to downstream auxin-response gene is micro-regulated and complex. In *Arabidopsis*, the plant height of *arf6/arf8* double knockout mutant decreased leaf shrinkage. Because of the redundant function of homologous ARFs, the plant height of single deletion mutant of *ARF6* and *ARF8* was less serious than that of double mutants [30]. To fully understand the function of ARFs in stem development, more mutants and species need to be analyzed. The expressions of *EXPA5* and *EXPA2*, *EXPA3*, *EXPA6* in *A. thaliana* are lower in *axr2/iaa7* mutant than 3529 indicated that they were also regulated by auxin signal (Figure S5b). The continuous expression of *BnaEXPA5* perhaps increased the plant height and leaf area size of transgenic *A. thaliana* (Figure 6) through its cell wall-loosening ability.

The cell wall not only provides mechanical strength for the plant body, but also physically controls many key features of growing plant cells, including growth, differentiation, size, shape, intercellular communication, water/turgor relations and defense against pathogens [13]. Cell wall can be divided into primary cell wall and secondary cell wall. Primary cell wall is a dynamic structure, which can support cell growth and expand with cell growth. Its expansion plays a decisive role in determining the morphology of plants. The secondary cell wall structure begins to form after cell growth, which can finally shape and support plants. The primary cell wall is composed of three distinctive polysaccharides (cellulose, hemicellulose, and pectin) and is usually organized into multilayer nanostructures, especially in the epidermal wall that physically protect and limit growth of leaves and stems [59,60]. In each layer, the cellulose fibrils are arranged in a common direction, forming a reticulated, noncovalent network, but the direction varies between the layers; hemicellulose bind noncovalently to cellulose, and well-hydrated pectins form a gel-like matrix hosting the stiff cellulose network [61]. However, the structure-function of primary plant cell walls is not well understood. The latest analysis of cell wall models shows that cellulose noncovalently binds together, providing stress-dependent elasticity, stiffening, and then slide over each other as the cell is stretched, thereby providing plasticity. Thus, cellulose is key to the cell wall’s strength, rigidity and plasticity [61]. Expansins were discovered in cell walls from cucumber as they responsible for wall extension [62]. Bundled regions where cellulose is in close physical contact with one another potentially function as sites of cell wall loosening and creep by expansins [60].

In the acid-growth hypothesis, auxin signaling induces expression of the gene encoding plasma membrane H^+^-ATpase proton pump, which pumps out H^+^ to the wall matrix, resulting in plasma membrane acidification (pH 4.5–6) [63]. Auxin-induced acidic pH is necessary to activate expansins, which cause the cellulose fibril–fibril sliding by acting on the cellulose bundled regions in a non-enzymatic manner, promoting wall loosening, hydration, and expansion [60,63]. In this study, the expansin protein encoding gene *BnaEXPA5* was significantly downregulated in the stem of the dwarf mutant *ndf-2*, and its expression could also be affected by exogenous auxin. BnaA03.IAA7- and BnaARF6/8-dependent auxin signaling can regulate the expression of *BnaEXPA5* gene. These results suggest that the phytohormone auxin not only activates proton and potassium channels, but also directly regulates the expression of genes encoding expansins, in the process of stimulating cell elongation via increasing cell wall extensibility.

**Figure 7:**
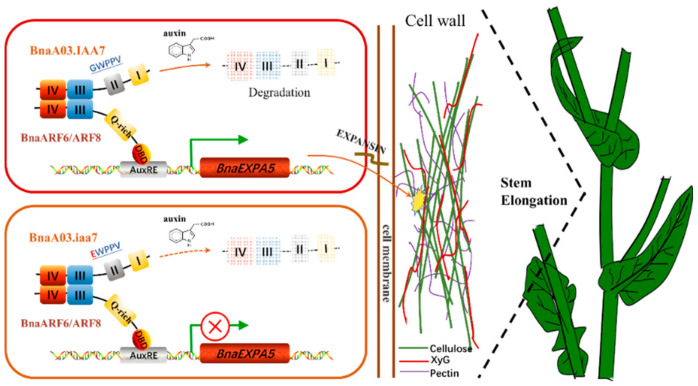
Model of stem elongation controlled by *BnaEXPA5* in *Brassica napus*. Under the appropriate concentration, auxin mediates the ubiquitination and degradation of BnaA03.IAA7 releasing its inhibition on BnaARF6 and BnaARF8. Then the transcription of *BnaEXPA5* is activated, and its protein products play the role of loosening the wall and promoting the process of plant stem elongation. In *ndf-2*, the mutated EWPPV motif of BnaA03.iaa7 prevents its degradation, and the inhibition on BnaARF6 and BnaARF8 continues, so the transcription of *BnaEXPA5* is blocked.

Based on the above results, we propose an auxin dependent model of stem and leaf development in *B. napus* (Figure 7). BnaA03.IAA7- and BnaARF6/8-dependent signaling pathways stimulate the transcription of *BnaEXPA5*, which in turn allows auxin responses, including cell expansion and stem elongation. However, we cannot rule out the possibility of other *IAAs*, *ARFs*, *EXPs* or other cell wall synthesis and modification-related genes participating in the regulation of these processes, and how auxin regulates cell wall modifications over time remains elusive. In addition, phytohormones closely related to plant height, such as GA and BR, may also be involved in this process. For example, the transcription factor Brassinazole Resistant 1 can regulate the expression of auxin response genes through the interaction between MR and ARF6 [64]. The interactions between inhibitors prevent ARF6 and ARF8 from binding to target DNA [65]. In conclusion, the Aux/IAA and ARF dependent signaling pathways can control cell expansion and stem elongation, which can make rape adapt to the environment.

## 4. Materials and Methods

### 4.1. Plant Materials and Growth Conditions

The wild-type background parent 3529 is a doubled gynogenetic haploid line of *Brassica napus* L. [66]. A double-low GMS line *156B* was developed by the Laboratory of Genetics, Department of Biology, Sichuan University. Seeds of 3529 were bombarded by fast neutrons and treated with diethyl sulphate (DES), as described by Zhao et al. [66]. After the treated seeds germinated, they were carefully transplanted into the cropland to obtain M_1_ plants. M_2_ seeds harvested from selfed M_1_ plants were mixed sown to form M_2_ population. Mutants were screened from M_2_ population and dwarfed seedlings were identified. After multiple generations of self-pollination and selection of dwarf plants, two genetically pure lines of dwarf mutants were obtained, one named *ndf-1*, described in previous reports [67], and another named *ndf-2*.

The average height of *ndf-1* was 73 cm, while the height of *ndf-2* was about 87 cm. The height of both *ndf-1* and *ndf-2* was significantly lower than that of its wild-type parent 3529 (about 196 cm). The cultivar *156B* with a similar height to 3529 was selected as control for genetic localization. All materials were grown on the research farm of Sichuan University and all farming operations were carried out according to conventional techniques. Plant height was measured from the ground to the top of the inflorescence at maturity. In laboratory experiments, seeds of rapeseeds or Arabidopsis thaliana were surface disinfected with 75% alcohol for 1 min, followed by 1% (*V*/*V*) sodium hypochlorite for 10 min, and then thoroughly rinsed with deionized water. Then all seeds were transferred to growth chambers (16 h light/8 h dark cycle at 23 °C). All wild and mutant *Arabidopsis* seeds were purchased from the Arabidopsis Biological Resource Center (ABRC).

### 4.2. Genetic Analysis

*ndf-2* was crossed with wild-type line 3529, and F_1_ plants self-crossed to obtain F_2_ seeds. Some F_1_ plants backcrossed with *ndf-2* and 3529 to obtain B_11_ and B_12_, respectively. All crosses were done by means of emasculation followed by artificial insemination. *ndf-2*, 3529, F_1_, F_2_, B_11_ and B_12_ were grown under strictly controlled and consistent conditions. Plant height was measured at maturity and plants’ classification was conducted according to the frequency distribution of plants with different heights in each population. Segregation ratios in the F_1_, F_2_, B_11_ and B_12_ populations were tested by Chi-squared (χ^2^) goodness-of-fit test. The populations of 3529, *ndf-2*, F_1_, F_2_, B_11_ and B_12_ contained 60, 60, 56, 576, 164 and 234 individuals, respectively.

### 4.3. Mapping and Identification of ndf-2 Gene

The F_2_ populations generated from the cross between *ndf-2* and *156B* were used to determine the position of *ndf-2*. Equal amounts DNA from 30 of 300 F_2_ plants with the extreme dwarf phenotype were mixed to form the dwarf bulk (Df-pool), and from 30 tall plants to form the wild-type bulk (WT-pool). Two parental pools (wild type, mutant) were composed of 20 individual DNA samples mixed equally using the same method for BSA-seq analysis as described by Abe et al. [68]. The constructed DNA libraries were sequenced on the Illumina sequencing platform by Genedenovo Biotechnology Co., Ltd. (Guangzhou, China). The clean reads from each sample were used to align against the *Brassica napus ZS11* reference genome. Variant calling was performed for multi-sample using the GATK software [69]. Sliding window analysis was applied to calculate the frequency distribution of SNP (SNP-index). The Δ(SNP-index) was obtained by subtracting the SNP-index of the WT-pool from the Df-pool. QTL was identified in positive or negative peak regions with a confidence interval of 95%. Then, SNPs and InDels in peak regions were selected for genotypes and annotation, and potential functional variations were screened.

Since functional mutations mainly originate from the coding gene region, priorities, mutations that directly affect the amino acid coding of the genes in peak regions were selected, include SNP and InDel mutations in upstream, UTR5, exonic, splicing region, UTR3 and downstream of genes, but synonymous mutations. Then, according to the genetic property of *ndf-2* gene, an approach was proposed to isolate causal mutation sites harbored in the peak regions. Mutations were excluded as follows:

- the SNPs and InDels are heterozygous in wild-type parent and WT-pool;
- the SNPs and InDels are inconsistent between wild-type parent and WT-pool;
- the SNPs and Indels are the same between Df-pool and WT-pool;
- the SNPs and Indels are heterozygous in mutant parent.

### 4.4. Transcriptome Analysis

The tissues of *ndf-2* and 3529 were used to extract RNA, including the roots and leaves at the seedling stage, the stems and leaves at the booting stage, the stems and leaves and flowers at the flowering stage, and siliques at the silique stage. Samples were collected and frozen in liquid nitrogen and stored at −80 °C for RNA preparation. For each sample, two biological replicates were conducted, and each biological replicate contains three individual plants. The integrity and purity of all RNA samples were detected by 1% agarose gel electrophoresis, A260/A280 value and A230/A280 value obtained from Nanodrop 2000 (Thermo Scientific). Illumina TruseqTM RNA Sample Prep Kit was used to construct the sequencing library, and the sequencing data was generated by Illumina HiSeq platform. Filtered clean reads were mapped to the reference genome of *Brassica napus* ‘*Darmor-bzh*’. The screening criteria for differentially expressed genes (DEGs) were: FDR < 0.05 and |log_2_FC| ≥ 3. FDR represents the false discovery rate. FC represents the ratio of FPKM between two samples.

### 4.5. Fluorescent Quantitative Analysis

The different tissues of rapeseeds and Arabidopsis used in this study, including roots, hypocotyls, stems and leaves, were frozen in liquid nitrogen. Total RNA was extracted from frozen tissue using RNAprep Pure Plant Kit (Tiangen, Beijing, China) according to the protocol. Using RNA as template, reverse transcription was performed using the One-Step gDNA Removal and cDNA Synthesis SuperMix (TransGen, Beijing, China). The gene transcription level was measured using the Top Green qPCR SuperMix (TransGen, Beijing, China) on the Fluorescence quantitative PCR system (Bio-Rad, Hercules, CA, USA). The transcript levels of endogenous genes *BnaActin* in *Brassica napus* and *AtUBQ5* in *Arabidopsis thaliana* were used as references to ensure equal RNA loading, respectively. The corresponding primer sequences used for qPCR analyses are shown in Table S1.

### 4.6. Yeast Two-Hybrid Screening Assay

For the interaction analysis of BnaA03.IAA7, BnaARF6 and BnaARF8, total RNA from the *Brassica napus* 3529 was used as templates to amplify the Open Reading Frame (ORFs) of *Bna IAA7* (*BnaA03.IAA7*, *BnaA05.IAA7*, *BnaC02.IAA7*, *BnaC07.IAA7*), *BnaARF6* (*BnaC05.ARF6*, *BnaA08.ARF6*) and *BnaARF8 (BnaA04.ARF8*, *BnaA07.ARF8)*. Then CDS of four *BnaIAA7s* were fused to the binding domain (BD) of pGBKT7 vector (Clontech, Mountain View, CA, USA) by enzyme digestion and enzyme ligation to construct pBD-IAA7 recombinant plasmid, respectively. BnaIAA7s and DNA binding domain of ARFs (ARF-DBD) and PB1 domain of ARFs (ARF-PB1) were cloned into pGADT7 vector (Clontech, Mountain View, CA, USA) to generate AD-IAA7, AD-ARF-DBD and AD-ARF-PB1 recombinant, respectively. AD- and BD-recombinant plasmids were transformed into AH109 yeast strains and then incubated at 30 °C on the control medium (SD/-Leu-Trp) plate for 3 days. The interactions were detected by spreading positive clones on the selective medium (SD/−Leu−Trp−His−Ade). The primers for PCR amplification and their corresponding restriction sites are listed in Table S1.

### 4.7. Hormone Treatment

The sterilized seeds were cold-treated for 3 days, then they were spotted on hormone-free Murashige and Skoog (MS) medium in a growth chamber at 23 °C and germinated vertically for 3 days. The growth consistent seedlings were transferred to square plates containing MS medium with or without different concentrations of IAA or NPA. For qPCR analysis, samples were taken after ten days of growth. Each treatment sets three repeats, and one repeat contains 3 plants. For the hormone response experiment, 1-week-old 3529 and *ndf-2* seedlings were treated with auxin (IAA; 5 μM), the polar auxin transport inhibitor N-1-naphthylphthalamic acid (NPA; 0.5 μM) or both for 20 days. The lengths of the hypocotyls and roots were then measured and photographed. Each treatment sets three repeats, and one repeat contains at least 3 plants. All the plants were grown under the same greenhouse conditions (23 °C, 16 h light/8 h dark)

### 4.8. Y1H Assays

The yeast one-hybrid assay was used to detect whether the BnaARF6 and BnaARF8 proteins can bind to the auxin response elements (AuxRE) on promoter of the encoding gene of cell wall loosening protein EXPANSIN A5 (EXPA5). The experiments were performed according to the protocol of Matchmaker Gold Yeast One-Hybrid Library Screening System (Clontech, USA). Target sequences are comprised of the AuxRE sequence of GAGACA (–1603 bp to –1608 bp) and 4 bp flanking sequences located in upstream of the EXPA5 gene start codon, then tandem three copies were generated by oligonucleotide synthesis. *HindIII* and *XhoI* restriction sites were designed at the ends of two antiparallel oligonucleotides to form overhanging sticky ends after annealing and were compatible with the sticky ends of the appropriately digested pAbAi Vector. The tandem 3 × AuxRE target sequence was inserted into the upstream of Aureobasidin A (AbA) resistance reporter gene (*AUR1*-*C*) in the pAbAi vector. A control with a base substitution in AuxRE (GAGACA, named mAuxRE) was produced by the same procedure as before. The recombinant plasmids were labeled as pBait-AuxRE and pBait-mAuxRE, respectively. The DNA binding domain of BnaARF6 and BnaARF8 (ARF-DBD) was fused with the activation domain of GAL4 protein in pGADT7 vector as described in the Y2H assays (labeled as AD-ARF-DBD). The primers are listed in Table S1.

The preparation and transformation of Y1HGold Yeast competent cells was performed according to The Yeastmaker Yeast Transformation System 2 protocol. *BstBI* enzyme was used to digest the pBait-AuxRE vector, and then the linearized plasmid was transformed into Y1HGold by homologous integration to produce the bait strains. Then the transformants were selected on SD/-Ura media. Colony PCR was used to confirm correct integration of the plasmid into the yeast genome. One colony from each confirmed bait strains was picked and resuspended with 0.9% NaCl, and the OD600 was adjusted to ~0.002. Then they were spread on SD/-Ura media containing Aureobasidin A (AbA) at different concentrations (100 ng/mL, 150 ng/mL, 200 ng/mL) to determine the minimum inhibitory concentration of AbA for the bait strain. Subsequently, AD-ARF-DBD plasmids were transformed into the bait strains, the transformants were selected on SD/-Leu media and the single colonies were cultured in YPDA medium. All yeast cultures were adjusted to the same OD600 value and diluted by gradient dilution method (1/10). Then the cultures were spotted on SD/-Ura, SD/-Leu, and SD/-Ura with 100 ng/mL AbA, and the growth of each yeast strains was observed and photographed after these plates were incubated at 30 °C for 2~3 days.

### 4.9. Development of AS-PCR Markers

A single base substitution occurred on the second exon of *BnaA03.IAA7* (*LOC106439612*) on chromosome A3 (located at 751 bp after the initial translation site), G in the wild type 3529 and A in *ndf-2* rapeseed. In order to detect the linkage relationship between the mutation and dwarfing traits, an Allele-specific PCR marker was designed and a single nucleotide artificial mismatch was introduced into the third base closest to the 3′ end (SNP site) of the upstream primer as the described method in the web protocol [70]. Using this pair of primers, a 798 bp DNA fragment can be amplified from the *ndf-2* genome, but not from 3529 or other wild types. The primers for this AS-PCR marker are listed in Table S1

### 4.10. Scanning Electron Microscopy

The hypocotyl, stem, and leaf vein of *Brassica napus* were carefully cut with a sharp blade. The segments were placed in a tube and submerged in 2.5% glutaraldehyde solution, fixed at room temperature for 2 h, and then transferred to a refrigerator at 4 °C for preservation. The samples were then dehydrated, dried with critical point drier (Quorum K850), sputter-coated with gold (HITACHI MC1000) and observed with a scanning electron microscope (HITACHI Regulus 8100). Cell size was measured and analyzed using image-pro plus 6.0 software.

### 4.11. Protoplast Transformation and Transient Expression Assays

The BnaA03.IAA7 full-length CDS was cloned into the vector pBI221-EGFP, the *EXPA5* full-length CDS was cloned into the vector pBI221-EGFP and pBI121-EGFP to generate GFP-IAA7, 221-GFP-EXPA5, and 121-GFP-EXPA5 plasmids for subcellular localization analysis, respectively. The pBI121-EGFP fusion proteins were transiently expressed in epidermal cells of tobacco (*Nicotiana benthamiana*) leaves induced by Agrobacterium tumefaciens GV3101. Tobacco protoplast isolation and transformation procedure was performed as described by Yoo [71]. GFP-IAA7 and 221-GFP-EXPA5 plasmids were transformed into tobacco protoplast and incubated for 18 h, then GFP signals were observed and photographed with a fluorescence microscope (Leica microsystems DM4 B).

### 4.12. Transgenic Arabidopsis Thaliana

The BnaEXPA5 full-length CDS was amplified from *Brassica napus* 3529 mRNA by PCR using primers EXPA5-CDS-F and EXPA5-CDS-R. PCR products with restriction site at both ends were cloned into the binary vector pFGC5941 to produce the constructs p35S::EXPA5 by the methods of enzyme digestion and enzyme ligation. The recombinant plasmid was transformed into agrobacterium tumefaciens GV3101 by heat shock method. Wild-type *Arabidopsis* was transformed using the Agrobacterium-mediated floral dip method [72]. Transgenic T1 was selected by spraying 1/1500 Basta solution onto the leaves. PCR identification of the surviving positive lines was performed using specific primers of Bar gene and EXPA5 gene. Ten positive lines that T2 generation, with a basta resistance separation ratio of 3:1, were selected to generate homozygous transgenic plants (T3) for further analysis. The primers are listed in Table S1. All the transgenic and wild type plants were grown in soil (peat:vermiculite = 3:1) under the same greenhouse conditions (23 °C, 16 h light/8 h dark).

### 4.13. Data Statistics and Analysis

Statistical analysis was performed using the SPSS software (IBM Corporation) and Origin 8.5 software (Origin Lab Corporation). Data were analyzed by one-way ANOVA and post hoc Tukey’s test. Significance was defined as *p* < 0.05.

## 5. Conclusions

In summary, a gain-of-function mutant *ndf-2* with dwarfism and wrinkled leaf was derived from fast neutron and DES mutagenized DH line 3529 (*Brassica napus*). The candidate region for dwarfism appears on the A03 chromosome of *Brassica napus L* through the BSA-seq method. Subsequently, according to the genetic property of *ndf-2*, SNPs and InDels in all the candidate regions from BSA-seq were screened and filtered. A total of 83 SNPs and InDels was retained, among which only six mutations were located in exons and distributed in six genes. Further analysis revealed that an amino acid substitution from G to E in in the conserved Degron motif GWPPV of BnaA03.IAA7 completely co-segregated with the dwarf phenotypes as demonstrated by the AS-PCR marker experiments. Since BnaA03.IAA7 encodes a transcriptional suppressor of auxin signal transduction, we also investigated the regulation of auxin-mediated stem elongation in rapeseeds. The transcription activators BnaARF6 and BnaARF8 were demonstrated as interactors of BnaA3.IAA7, the gene *BnaEXPA5* encoding cell wall loosening protein as a common target of BnaARF6/8. In this pathway, BnaA03.IAA7 inhibits the transcriptional activity of BnaARF6/8, and the gain-of-function mutation prevents its degradation induced by auxin, which in turn prevents BnaARF6/8 from activating the expression of downstream *BnaEXPA5* gene. The downregulation of *BnaEXPA5* expression eventually results in inhibition of cell wall loosening and leading to dwarfing phenotype. Our findings identified one allele of BnaA03.IAA7 responsible for plant dwarf phenotype and provided new insights for a better understanding of the molecular mechanisms underlying cell wall expansion mediated stem elongation and plant architecture. Furthermore, the manipulation of IAA, ARF and expansins combined with other breeding tools and biotechnology, such as new genome editing techniques, can be a useful strategy for improving our crops, especially in plant type and resistance. However, the regulation necessary to produce distinct and dynamic auxin output is multi-level, the future interest and challenge will be to uncover the details involved in the auxin signaling network and understand the importance of overlapping functions and compensation of complex regulatory systems. In addition, due to the complexity of plant cell wall and the lack of a deeper understanding of how cell wall polymers are assembled to form a load bearing, but extensible, primary cell wall, more research is needed to reveal the complex mechanism of cell wall and the mystery of expansin loosening.

## Figures and Tables

**Table 1:** Principal agronomic characteristics of two dwarf mutants.

Line	Height (cm)	PPEB (cm)	LMI (cm)	NPB	NSB	NPPP	NSPP	WS (g)	YPP (g)
*ndf-1*	71.4 ± 3.3	5.8 ± 2.7	8.2 ± 3.2	6.6 ± 2.4	5.5 ± 2.2	237.6 ± 55.7	11.8 ± 3.7	3.6 ± 0.2	10.3 ± 2.6
*ndf-2*	86.8 ± 4.9	7.1 ± 2.3	11.0 ± 2.2	7.1 ± 1.8	5.8 ± 1.7	256.1 ± 41.4	10.6 ± 4.1	3.5 ± 0.3	12.9 ± 3.5
3529 (ck)	198.7 ± 15.9	22.8 ± 5.2	67.8 ± 8.4	9.1 ± 2.3	14.7 ± 1.3	388.0 ± 35.8	17.5 ± 2.9	3.4 ± 0.2	23.4 ± 4.3

Data in the table are mean ± SD. PPEB, place of primary effective branches; LMI, length of main inflorescence; NPB, no. of primary branches; NSB, no. of secondary branches; NPPP, no. of effective pods per plant; NSPP, no. of seeds per pod; WS, weight of 1000 seeds; YPP, yield per plant.

**Table 2:** Plant heights and Chi-squared test for the segregation ratio of dwarfism.

Population	Number of Plants (Height Range)	Segregation Ratio			Expected Ratio	χ^2^	*p*
		Tall	Semi-Dwarf	Dwarf			
3529	60 (172–213 cm)	60	0	0	1:0:0	-	-
*ndf-2*	60 (73–95 cm)	0	0	60	0:0:1	-	-
F_1_	56 (121–167 cm)	0	56	0	0:1:0	-	-
F_2_	576 (47–219 cm)	139 (≥170 cm)	268 (110–170 cm)	159 (≤110 cm)	1:2:1	3.125	>0.05
B_11_ (F_1_ × 3529)	164 (115–215 cm)	73 (≥170 cm)	91 (110–170 cm)	0 (≤110 cm)	1:1:0	1.97	>0.05
B_12_ (F_1_ × *ndf-2*)	234 (56–164 cm)	0 (≥170 cm)	127 (110–170 cm)	107 (≤110 cm)	0:1:1	1.71	>0.05

## Data Availability

Not applicable.

## Supplementary Materials

The following are available online at https://www.mdpi.com/article/10.3390/ijms22169018/s1.

## Abbreviations

ARF	Auxin response factor
Aux/IAA	Auxin/Indole-3-Acetic Acid
BC	Back cross
BR	Brassinosteroid
BSA-seq	Bulked Segregant Analysis-sequencing
DEGs	differential expression genes
DES	Diethyl sulfate
DH	Double haploid
FPKM	Fragments Per Kilobase of exon model per Million mapped fragments
GA	Gibberellin
IAA	Indole-3-Acetic
InDel	Insertion-deletion
NPA	1-naphthylphthalamic acid
SNP	Single nucleotide polymorphism
EXP	EXPANSIN
qRT-PCR	Quantitative reverse transcription PCR

## References

[1] Khush G.S. (1995). Breaking the yield frontier of rice. GeoJournal.

[2] Islam N., Evans E.J. (1994). Influence Of Lodging and Nitrogen Rate on the Yield And Yield Attributes Of Oilseed Rape (*Brassica-Napus* L). Theor. Appl. Genet..

[3] Khush G.S. (2001). Green revolution: The way forward. Nat. Rev. Genet..

[4] Fernandez M.G.S., Becraft P.W., Yin Y.H., Lubberstedt T. (2009). From dwarves to giants? Plant height manipulation for biomass yield. Trends Plant Sci..

[5] Ashikari M., Sasaki A., Ueguchi-Tanaka M., Itoh H., Nishimura A., Datta S., Ishiyama K., Saito T., Kobayashi M., Khush G.S. (2002). Loss-of-function of a rice gibberellin biosynthetic gene, GA20 oxidase (GA20ox-2), led to the rice ‘green revolution’. Breed. Sci..

[6] Ueguchi-Tanaka M., Fujisawa Y., Kobayashi M., Ashikari M., Iwasaki Y., Kitano H., Matsuoka M. (2000). Rice dwarf mutant d1, which is defective in the alpha subunit of the heterotrimeric G protein, affects gibberellin signal transduction. Proc. Natl. Acad. Sci. USA.

[7] Tanabe S., Ashikari M., Fujioka S., Takatsuto S., Yoshida S., Yano M., Yoshimura A., Kitano H., Matsuoka M., Fujisawa Y. (2005). A novel cytochrome P450 is implicated in brassinosteroid biosynthesis via the characterization of a rice dwarf mutant, dwarf11, with reduced seed length. Plant Cell.

[8] Lin H., Wang R.X., Qian Q., Yan M.X., Meng X.B., Fu Z.M., Yan C.Y., Jiang B., Su Z., Li J.Y. (2009). DWARF27, an Iron-Containing Protein Required for the Biosynthesis of Strigolactones, Regulates Rice Tiller Bud Outgrowth. Plant Cell.

[9] Mai Y.X., Wang L., Yang H.Q. (2011). A Gain-of-Function Mutation in IAA7/AXR2 Confers Late Flowering under Short-day Light in Arabidopsis. J. Integr. Plant. Biol..

[10] Nagpal P., Walker L.M., Young J.C., Sonawala A., Timpte C., Estelle M., Reed J.W. (2000). AXR2 encodes a member of the Aux/IAA protein family. Plant. Physiol..

[11] Wang Y.H., Li J.Y. (2008). Molecular basis of plant architecture. Annu. Rev. Plant Biol..

[12] Yamamuro C., Ihara Y., Wu X., Noguchi T., Fujioka S., Takatsuto S., Ashikari M., Kitano H., Matsuoka M. (2000). Loss of function of a rice brassinosteroid insensitive1 homolog prevents internode elongation and bending of the lamina joint. Plant Cell.

[13] Cosgrove D.J. (2005). Growth of the plant cell wall. Nat. Rev. Mol. Cell Biol..

[14] Cosgrove D.J. (2014). Re-constructing our models of cellulose and primary cell wall assembly. Curr. Opin. Plant Biol..

[15] Bashline L., Lei L., Li S.D., Gu Y. (2014). Cell Wall, Cytoskeleton, and Cell Expansion in Higher Plants. Mol. Plant.

[16] Ba L.J., Shan W., Kuang J.F., Feng B.H., Xiao Y.Y., Lu W.J., Chen J.Y. (2014). The Banana MaLBD (LATERAL ORGAN BOUNDARIES DOMAIN) Transcription Factors Regulate EXPANSIN Expression and Are Involved in Fruit Ripening. Plant Mol. Biol. Rep..

[17] Rauf M., Arif M., Fisahn J., Xue G.P., Balazadeh S., Mueller-Roeber B. (2013). NAC Transcription Factor SPEEDY HYPONASTIC GROWTH Regulates Flooding-Induced Leaf Movement in Arabidopsis. Plant Cell.

[18] Hur Y.S., Um J.H., Kim S., Kim K., Park H.J., Lim J.S., Kim W.Y., Jun S.E., Yoon E.K., Lim J. (2015). Arabidopsis thaliana homeobox 12 (ATHB12), a homeodomain-leucine zipper protein, regulates leaf growth by promoting cell expansion and endoreduplication. New Phytol..

[19] Ribas A.F., Silva N.V.E., dos Santos T.B., Abrantes F.L., Custodio C.C., Machado-Neto N.B., Vieira L.G.E. (2019). Regulation of alpha-expansins genes in Arabidopsis thaliana seeds during post-osmopriming germination. Physiol. Mol. Biol. Plants.

[20] Perrot-Rechenmann C. (2010). Cellular Responses to Auxin: Division versus Expansion. Cold Spring Harb. Perspect. Biol..

[21] Liscum E., Reed J.W. (2002). Genetics of Aux/IAA and ARF action in plant growth and development. Plant. Mol. Biol..

[22] Mockaitis K., Estelle M. (2008). Auxin Receptors and Plant Development: A New Signaling Paradigm. Annu. Rev. Cell Dev. Biol..

[23] Nishitani K., Masuda Y. (1981). Auxin-Induced Changes in the Cell-Wall Structure—Changes in the Sugar Compositions, Intrinsic-Viscosity and Molecular-Weight Distributions of Matrix Polysaccharides of the Epicotyl Cell-Wall of Vigna-Angularis. Physiol. Plant..

[24] Nemhauser J.L., Hong F.X., Chory J. (2006). Different plant hormones regulate similar processes through largely nonoverlapping transcriptional responses. Cell.

[25] Ren H., Gray W.M. (2015). SAUR Proteins as Effectors of Hormonal and Environmental Signals in Plant Growth. Mol. Plant.

[26] Kitomi Y., Inahashi H., Takehisa H., Sato Y., Inukai Y. (2012). OsIAA13-mediated auxin signaling is involved in lateral root initiation in rice. Plant Sci..

[27] Cheng H.T., Jin F.W., Zaman Q.U., Ding B.L., Hao M.Y., Wang Y., Huang Y., Wells R., Dong Y., Hu Q. (2019). Identification of Bna.IAA7.C05 as allelic gene for dwarf mutant generated from tissue culture in oilseed rape. BMC Plant Biol..

[28] Zheng M., Hu M.L., Yang H.L., Tang M., Zhang L., Liu H.F., Li X.K., Liu J.L., Sun X.C., Fan S.H. (2019). Three BnaIAA7 homologs are involved in auxin/brassinosteroid-mediated plant morphogenesis in rapeseed (*Brassica napus* L.). Plant Cell Rep..

[29] Oh E., Zhu J.Y., Bai M.Y., Arenhart R.A., Sun Y., Wang Z.Y. (2014). Cell elongation is regulated through a central circuit of interacting transcription factors in the Arabidopsis hypocotyl. eLife.

[30] Nagpal P., Ellis C.M., Weber H., Ploense S.E., Barkawi L.S., Guilfoyle T.J., Hagen G., Alonso J.M., Cohen J.D., Farmer E.E. (2005). Auxin response factors ARF6 and ARF8 promote jasmonic acid production and flower maturation. Development.

[31] Wu M.F., Tian Q., Reed J.W. (2006). Arabidopsis microRNA167 controls patterns of ARF6 and ARF8 expression, and regulates both female and male reproduction. Development.

[32] Kuluev B.R., Knyazev A.B., Lebedev Y.P., Chemeris A.V. (2012). Morphological and physiological characteristics of transgenic tobacco plants expressing expansin genes: AtEXP10 from Arabidopsis and PnEXPA1 from poplar. Russ. J. Plant Physiol..

[33] Kuluev B.R., Safiullina M.G., Knyazev A.V., Chemeris A.V. (2013). Effect of ectopic expression of NtEXPA5 gene on cell size and growth of organs of transgenic tobacco plants. Russ. J. Dev. Biol..

[34] Ma N.N., Wang Y., Qiu S.C., Kang Z.H., Che S.G., Wang G.X., Huang J.L. (2013). Overexpression of OsEXPA8, a Root-Specific Gene, Improves Rice Growth and Root System Architecture by Facilitating Cell Extension. PLoS ONE.

[35] Cho H.T., Cosgrove D.J. (2002). Regulation of root hair initiation and expansin gene expression in Arabidopsis. Plant Cell.

[36] Yu Z.M., Kang B., He X.W., Lv S.L., Bai Y.H., Ding W.N., Chen M., Cho H.T., Wu P. (2011). Root hair-specific expansins modulate root hair elongation in rice. Plant J..

[37] Zou H.Y., Wenwen Y.H., Zang G.C., Kang Z.H., Zhang Z.Y., Huang J.L., Wang G.X. (2015). OsEXPB2, a beta-expansin gene, is involved in rice root system architecture. Mol. Breed..

[38] Lee H.W., Kim J. (2013). EXPANSINA17 Up-Regulated by LBD18/ASL20 Promotes Lateral Root Formation During the Auxin Response. Plant. Cell Physiol..

[39] Minoia S., Boualem A., Marcel F., Troadec C., Quemener B., Cellini F., Petrozza A., Vigouroux J., Lahaye M., Carriero F. (2016). Induced mutations in tomato SlExp1 alter cell wall metabolism and delay fruit softening. Plant Sci..

[40] Esmon C.A., Tinsley A.G., Ljung K., Sandberg G., Hearne L.B., Liscum E. (2006). A gradient of auxin and auxin-dependent transcription precedes tropic growth responses. Proc. Natl. Acad. Sci. USA.

[41] Pelletier S., Van Orden J., Wolf S., Vissenberg K., Delacourt J., Ndong Y.A., Pelloux J., Bischoff V., Urbain A., Mouille G. (2010). A role for pectin de-methylesterification in a developmentally regulated growth acceleration in dark-grown Arabidopsis hypocotyls. New Phytol..

[42] Fu T., Zhou Y. (2013). Progress and future development of hybrid rapeseed in China. Eng. Sci..

[43] Li H., Li J., Song J., Zhao B., Guo C., Wang B., Zhang Q., Wang J., King G.J., Liu K. (2019). An auxin signaling gene BnaA3.IAA7 contributes to improved plant architecture and yield heterosis in rapeseed. New Phytol..

[44] Reed J.W., Wu M.F., Reeves P.H., Hodgens C., Yadav V., Hayes S., Pierik R. (2018). Three Auxin Response Factors Promote Hypocotyl Elongation. Plant Physiol..

[45] Calderon-Villalobos L.I., Tan X., Zheng N., Estelle M. (2010). Auxin Perception-Structural Insights. Cold Spring Harb. Perspect. Biol..

[46] Ramos J.A., Zenser N., Leyser O., Callis J. (2001). Rapid degradation of auxin/indoleacetic acid proteins requires conserved amino acids of domain II and is proteasome dependent. Plant Cell.

[47] Zenser N., Dreher K.A., Edwards S.R., Callis J. (2003). Acceleration of Aux/IAA proteolysis is specific for auxin and independent of AXR1. Plant J..

[48] Gray W.M., Kepinski S., Rouse D., Leyser O., Estelle M. (2001). Auxin regulates SCFTIR1-dependent degradation of AUX/IAA proteins. Nature.

[49] Worley C.K., Zenser N., Ramos J., Rouse D., Leyser O., Theologis A., Callis J. (2000). Degradation of Aux/IAA proteins is essential for normal auxin signalling. Plant J..

[50] Zenser N., Ellsmore A., Leasure C., Callis J. (2001). Auxin modulates the degradation rate of Aux/IAA proteins. Proc. Natl. Acad. Sci. USA.

[51] Dreher K.A., Brown J., Saw R.E., Callis J. (2006). The Arabidopsis Aux/IAA protein family has diversified in degradation and auxin responsiveness. Plant Cell.

[52] Havens K.A., Guseman J.M., Jang S.S., Pierre-Jerome E., Bolten N., Klavins E., Nemhauser J.L. (2012). A Synthetic Approach Reveals Extensive Tunability of Auxin Signaling. Plant Physiol..

[53] Moss B.L., Mao H.B., Guseman J.M., Hinds T.R., Hellmuth A., Kovenock M., Noorassa A., Lanctot A., Villalobos L.I.A.C., Zheng N. (2015). Rate Motifs Tune Auxin/Indole-3-Acetic Acid Degradation Dynamics. Plant Physiol..

[54] Timpte C., Wilson A.K., Estelle M. (1994). The axr2-1 mutation of Arabidopsis thaliana is a gain-of-function mutation that disrupts an early step in auxin response. Genetics.

[55] Uehara T., Okushima Y., Mimura T., Tasaka M., Fukaki H. (2008). Domain II Mutations in CRANE/IAA18 Suppress Lateral Root Formation and Affect Shoot Development in Arabidopsis thaliana. Plant Cell Physiol..

[56] Korasick D.A., Westfall C.S., Lee S.G., Nanao M.H., Dumas R., Hagen G., Guilfoyle T.J., Jez J.M., Strader L.C. (2014). Molecular basis for AUXIN RESPONSE FACTOR protein interaction and the control of auxin response repression. Proc. Natl. Acad. Sci. USA.

[57] Nanao M.H., Vinos-Poyo T., Brunoud G., Thevenon E., Mazzoleni M., Mast D., Laine S., Wang S.C., Hagen G., Li H.B. (2014). Structural basis for oligomerization of auxin transcriptional regulators. Nat. Commun..

[58] Powers S.K., Strader L.C. (2020). Regulation of auxin transcriptional responses. Dev. Dynam..

[59] Lipowczan M., Borowska-Wykret D., Natonik-Bialon S., Kwiatkowska D. (2018). Growing cell walls show a gradient of elastic strain across their layers. J. Exp. Bot..

[60] Cosgrove D.J. (2018). Nanoscale structure, mechanics and growth of epidermal cell walls. Curr. Opin. Plant Biol..

[61] Zhang Y., Yu J.Y., Wang X., Durachko D.M., Zhang S.L., Cosgrove D.J. (2021). Molecular insights into the complex mechanics of plant epidermal cell walls. Science.

[62] McQueen-Mason S., Durachko D.M., Cosgrove D.J. (1992). Two endogenous proteins that induce cell wall extension in plants. Plant Cell.

[63] Majda M., Robert S. (2018). The Role of Auxin in Cell Wall Expansion. Int. J. Mol. Sci..

[64] Liu K., Li Y.H., Chen X.N., Li L.J., Liu K., Zhao H.P., Wang Y.D., Han S.C. (2018). ERF72 interacts with ARF6 and BZR1 to regulate hypocotyl elongation in Arabidopsis. J. Exp. Bot..

[65] Ben-Targem M., Ripper D., Bayer M., Ragni L. (2021). Auxin and gibberellin signaling cross-talk promotes hypocotyl xylem expansion and cambium homeostasis. J. Exp. Bot..

[66] Zhao Y., Wang M.L., Zhang Y.Z., Du L.F., Pan T. (2000). A chlorophyll-reduced seedling mutant in oilseed rape, Brassica napus, for utilization in F-1 hybrid production. Plant Breed..

[67] Wang M.L., Zhao Y., Chen F., Yin X.C. (2004). Inheritance and potentials of a mutated dwarfing gene ndf1 in Brassica napus. Plant Breed..

[68] Abe A., Kosugi S., Yoshida K., Natsume S., Takagi H., Kanzaki H., Matsumura H., Yoshida K., Mitsuoka C., Tamiru M. (2012). Genome sequencing reveals agronomically important loci in rice using MutMap. Nat. Biotechnol..

[69] McKenna A., Hanna M., Banks E., Sivachenko A., Cibulskis K., Kernytsky A., Garimella K., Altshuler D., Gabriel S., Daly M. (2010). The Genome Analysis Toolkit: A MapReduce framework for analyzing next-generation DNA sequencing data. Genome Res..

[70] Liu J., Huang S.M., Sun M.Y., Liu S.Y., Liu Y.M., Wang W.X., Zhang X.R., Wang H.Z., Hua W. (2012). An improved allele-specific PCR primer design method for SNP marker analysis and its application. Plant Methods.

[71] Im J.H., Yoo S.D. (2014). Transient expression in Arabidopsis leaf mesophyll protoplast system for cell-based functional analysis of MAPK cascades signaling. Methods Mol. Biol..

[72] Clough S.J., Bent A.F. (1998). Floral dip: A simplified method for Agrobacterium-mediated transformation of Arabidopsis thaliana. Plant J..

