# Supplementary material for: A Gain-of-Function Mutant of IAA7 Inhibits Stem Elongation by Transcriptional Repression of EXPA5 Genes in Brassica napus
Source: Int J Mol Sci. 2021 Aug 21;22(16):9018. doi: 10.3390/ijms22169018 (PMC8396470; doi:10.3390/ijms22169018)
Supplement: Supplementary file 1 [file ijms-22-09018-s001.zip › Supplemental Figures.pdf]

Supplementary Information for

This file includes:

Supplementary text (Materials and Methods)

References for SI reference citations

Supplemental Figures S1 to S8

Supplemental Figures

Figure. S1 SNP-index and Δ(SNP-index) graphs from the BSA-seq analysis.

Figure.S2 Subcellular localization of BnaA03.IAA7 protein in leaf protoplasts of tobacco (*Nicotiana benthamiana*)

Figure. S3 Venn diagrams of up-regulated or down-regulated genes in RNA-seq analyses.

Figure. S4 Response of Brassica napus to Auxin.

Figure. S5 Arabidopsis iaa7 mutant.

Figure. S6 Subcellular localization of BnaC09.EXPA5.

Figure. S7 Yeast-two-hybrid assay for the interaction between BnaIAA7 proteins and BnaARF6/8 or BnaIAA7 proteins.

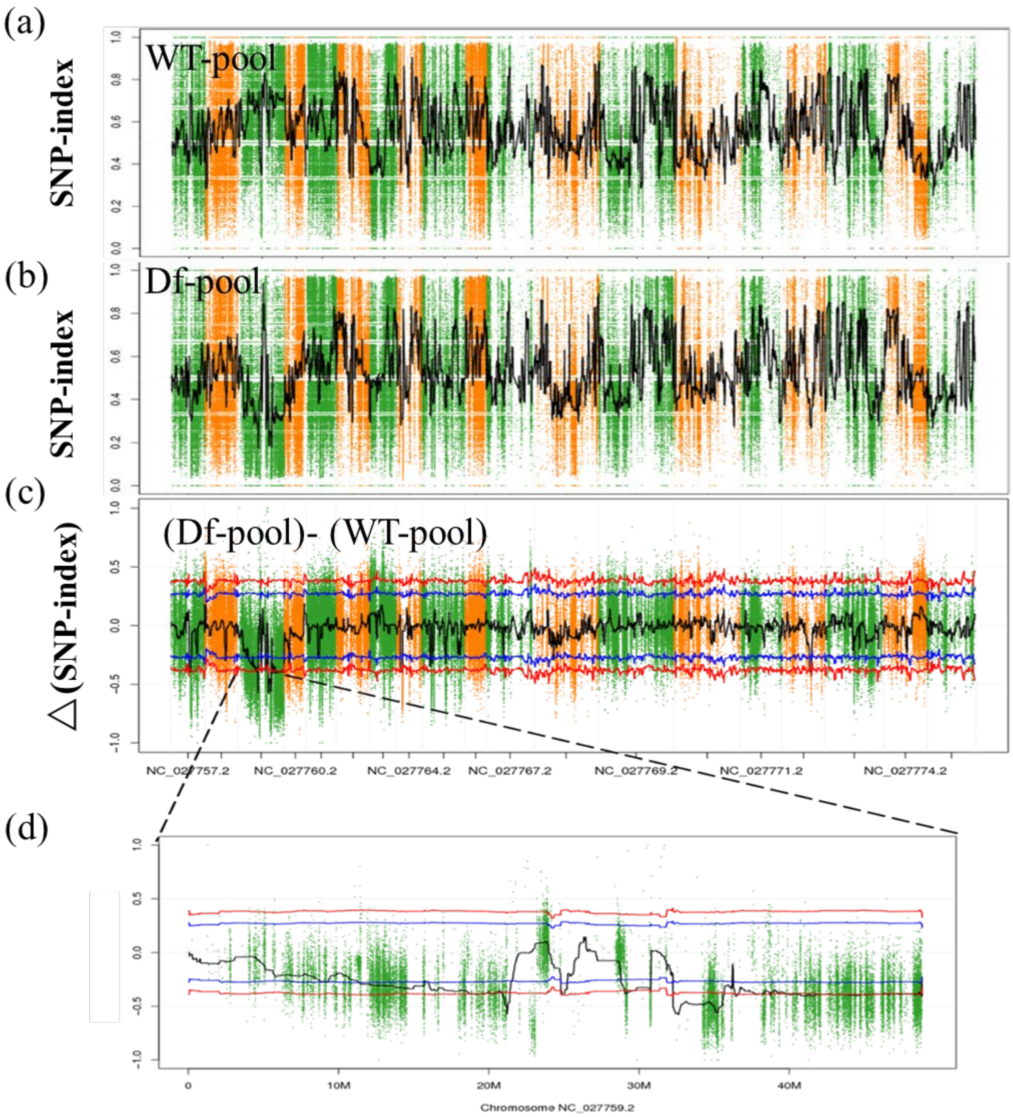

Figure S1. SNP-index and Δ(SNP-index) graphs from the BSA-seq analysis. The *x*-axis represents the physical position (Mb) of the 19 chromosomes in Brassica napus. The *y*-axis represents the SNP-index (a, b) or Δ(SNP-index) (c), which was calculated according to a 2000kb window with a 20kb sliding step. The Δ(SNP-index) was plotted by subtracting the WT-pool from the Df-pool. blue and red lines indicate the 95% ($p < 0.05$) and 99% confidence level ($p < 0.01$). (d) The candidate peak region harboring the *NDF2* located on chromosome A03 (accession:NC_027759.2).

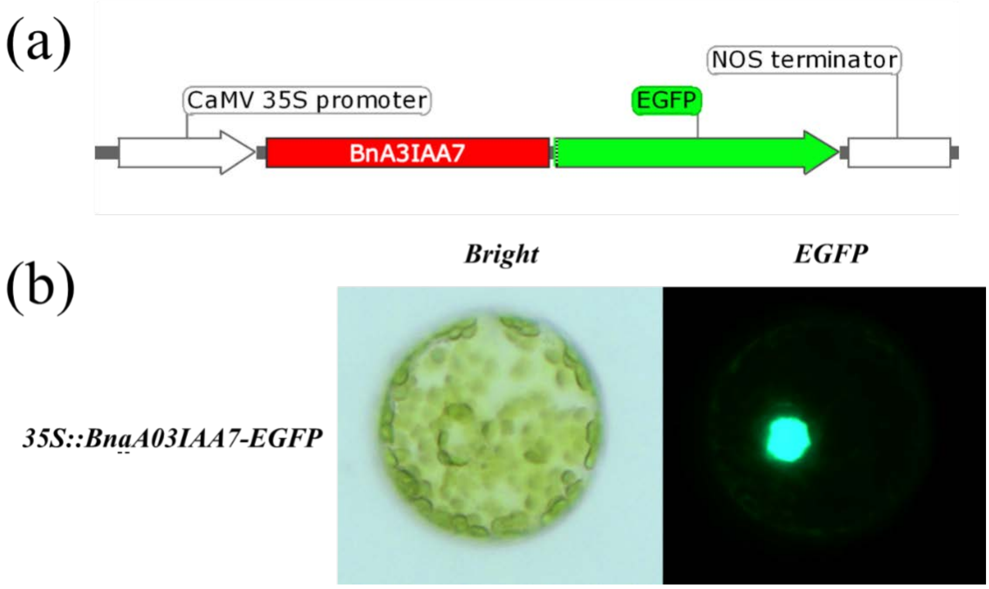

Figure S2. Subcellular localization of BNAA3IAA7 protein in leaf protoplasts of tobacco (Nicotiana benthamiana). (a) Schematic diagram of recombinant protein expression vector. (b) Fluorescence imaging of fusion proteins.

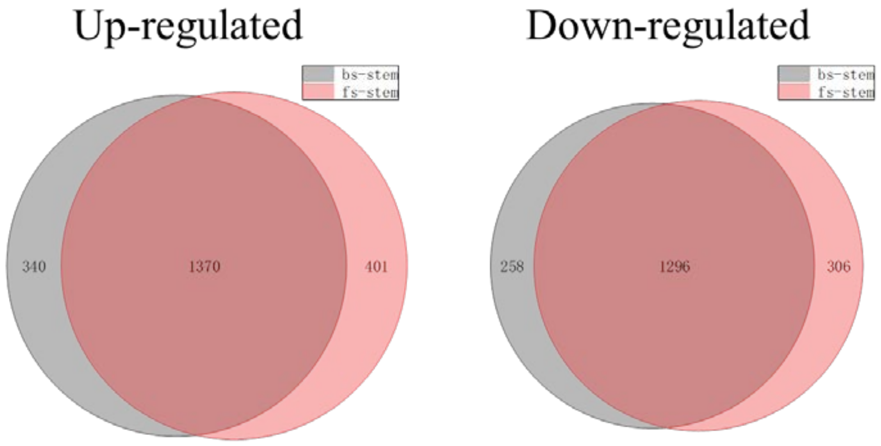

Figure. S3 Venn diagrams of up-regulated or down-regulated genes in RNA-seq analyses. Number of genes whose FPKM value were 8.0-fold higher or lower (FDR value < 0.05) in the NDF-2 when compared with 3529 in stem at bolting stage and flowering stage (bs-stem and fs-stem) were considered to be up-regulated or down-regulated.

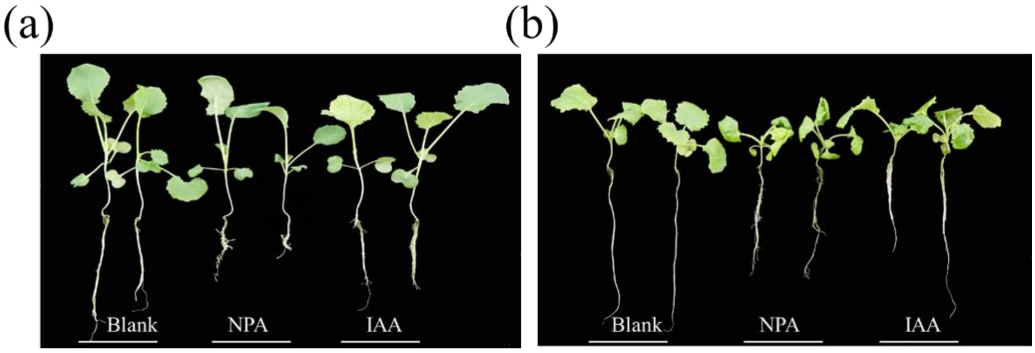

Figure. S4 Response of Brassica napus to Auxin. The morphology of 3529 (a) and NDF-2 (b) under IAA and NPA treatment.

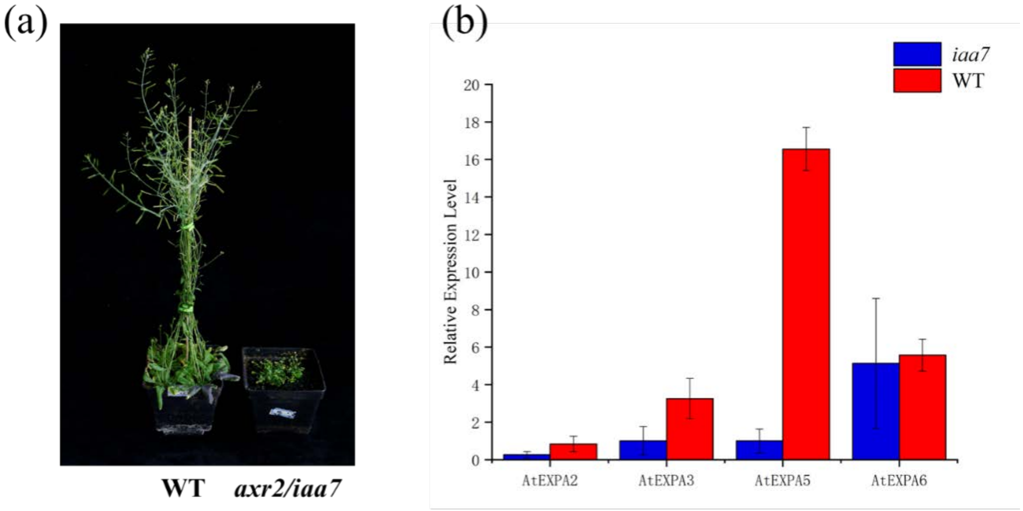

Figure. S5 Arabidopsis *iaa7* mutant. (a) Morphological differences between IAA7 mutants and WT (wild type). (b) The relative expression level of AtEXPA2(AT5G05290), AtEXPA3 (AT2G37640), AtEXPA5 (AT3G29030), AtEXPA6 (AT2G28950) gene in IAA7 and wild-type

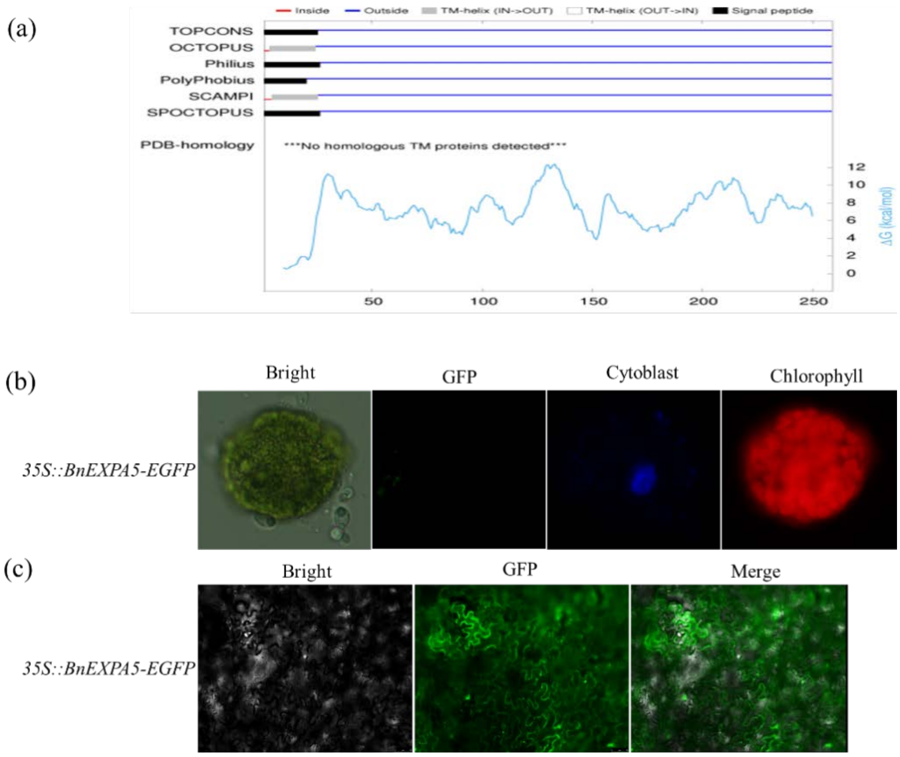

Figure. S6 Subcellular localization of BnaEXPA5. (a) Prediction of subcellular localization of BNAexpa5 protein by TOPCONS online software. The *x*-axis indicates the position of amino acid residues of BnaEXPA5, *y*-axis indicates the predicted location. (b, c)Fluorescence observation of bNAExPA5-EGFP fusion protein in tobacco protoplasts (b) and epidermal cells (c)

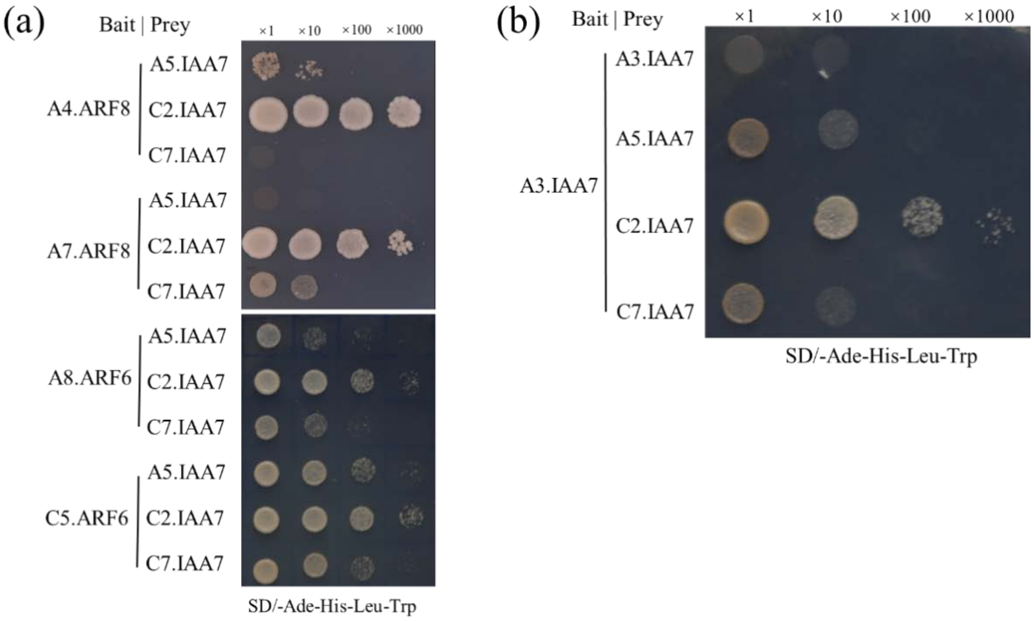

Figure. S7 Yeast-two-hybrid assay for the interaction between BnaIAA7 proteins and BnaARF6/8 or BnaIAA7 proteins. (a) Interaction between four ARF proteins and three other IAA7 homologues in Brassica napus L. (b) Interaction between BnaA03.IAA7 proteins and three other

IAA7 homologues. Yeast transformants were spotted on control medium (SD/-Leu-Trp) and selective medium (SD/-Ade-His-Leu-Trp) and the colonies were photographed at 3 days after the spotting

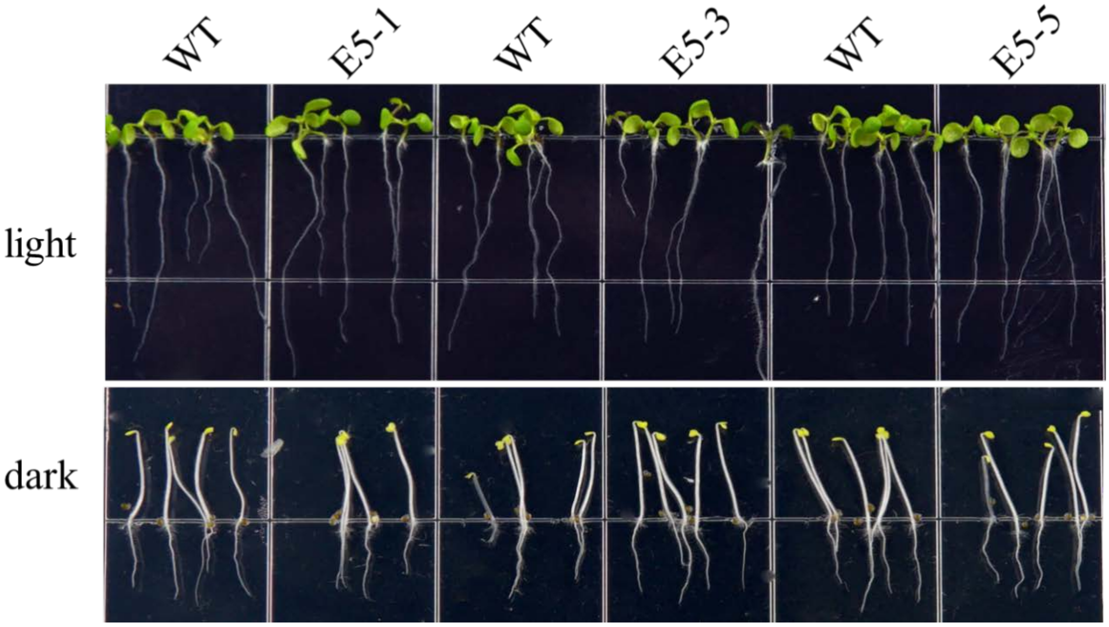

Figure. S7 Seedlings of three transgenic families and WT (wild-type) were grown on MS medium under light and dark conditions. The plates were placed vertically in an incubator at 23℃ with 16 h light/8 h darkness and the dark group was tightly wrapped with tin foil , photographed on the 5th day after sowing.
